# The mechanisms of genome-wide target gene regulation by TCF7L2 in liver cells

**DOI:** 10.1093/nar/gku1225

**Published:** 2014-11-20

**Authors:** Luke Norton, Xi Chen, Marcel Fourcaudot, Nikhil K. Acharya, Ralph A. DeFronzo, Sami Heikkinen

**Affiliations:** 1 Diabetes Division, University of Texas Health Science Center, San Antonio, TX 78229, USA; 2 Institute of Biomedicine, University of Eastern Finland, Kuopio 70211, Finland

## Abstract

In the liver Wnt-signaling contributes to the metabolic fate of hepatocytes, but the precise role of the TCF7L2 in this process is unknown. We employed a temporal RNA-Seq approach to examine gene expression 3–96 h following *Tcf7l2* silencing in rat hepatoma cells, and combined this with ChIP-Seq to investigate mechanisms of target gene regulation by TCF7L2. Silencing *Tcf7l2* led to a time-dependent appearance of 406 differentially expressed genes (DEGs), including key regulators of cellular growth and differentiation, and amino acid, lipid and glucose metabolism. Direct regulation of 149 DEGs was suggested by strong proximal TCF7L2 binding (peak proximity score > 10) and early mRNA expression changes (≤18 h). Indirect gene regulation by TCF7L2 likely occurred via alternate transcription factors, including *Hnf4a*, *Foxo1*, *Cited2*, *Myc* and *Lef1*, which were differentially expressed following *Tcf7l2* knock-down. *Tcf7l2*-silencing enhanced the expression and chromatin occupancy of HNF4α, and co-siRNA experiments revealed that HNF4α was required for the regulation of a subset of metabolic genes by TCF7L2, particularly those involved in lipid and amino-acid metabolism. Our findings suggest TCF7L2 is an important regulator of the hepatic phenotype, and highlight novel mechanisms of gene regulation by TCF7L2 that involve interplay between multiple hepatic transcriptional pathways.

## INTRODUCTION

The Wnt/β–catenin pathway is integral to embryonic development, cellular fate and migration, tumorigenesis and organogenesis (1). The major downstream transcriptional effectors of Wnt signaling are the lymphoid enhancer factor 1 (LEF1) and the T-cell factor (TCF) family of high-mobility group box (HMG-box)-containing proteins. These transcription factors bind DNA directly and recruit multiple transcription factors which, depending on the cellular context, activate or repress gene expression (2). Thus, the expression of Wnt target genes is highly coordinated and is controlled in a temporal and cell-specific manner, which is likely dependent on the expression, DNA binding and transcriptional activity of TCFs.

A good example of carefully coordinated Wnt signaling is during liver hepatocyte development. The liver is unique in that amongst all tissues in the human body, it regulates a large spectrum of metabolic pathways and plays a central role in the coordination of whole-body homeostasis. It is critical for protein, carbohydrate, lipid and drug metabolism, as well as hormone and bile production and detoxification (3). To accommodate this array of metabolic functions, the liver displays a remarkable degree of heterogeneity and functional plasticity, known as metabolic zonation (4). The zonation of the liver is transcriptionally controlled by Wnt signaling (5), and several studies have suggested that the transcriptional co-activator β-catenin is the master-regulator of liver zonation (6). However, what role individual TCFs play in determining the metabolic phenotype of hepatocytes is still largely unknown.

As a key member of the TCF family of transcription factors, TCF7L2 (transcription factor 7-like 2) is unique in that its gene is the most significant type 2 diabetes (T2DM) candidate gene identified to date (7), and recently attention has focused on the molecular and physiological role of TCF7L2 in the tissues relevant to T2DM pathogenesis—most notably the liver (8–10), brain (11), fat (12) and the pancreas (13–15). In the liver, several groups have examined the role of TCF7L2 in glucose metabolism and hepatic glucose production (HGP). In cultured rat, human and mouse hepatocytes, it has been shown that wild-type TCF7L2 negatively regulates gluconeogenesis (8,9,16). Silencing of TCF7L2 protein levels in these cells leads to an increase in glucose output associated with elevated expression of multiple gluconeogenic genes (8,9,16). In rodents, Oh *et al.* (10) first demonstrated that mice with reduced hepatic TCF7L2 levels similarly have elevated rates of HGP and higher fasting and postprandial plasma glucose levels *in vivo*. Although a recent study demonstrated that tamoxifen-induced liver TCF7L2 knock-out lowered fasting glucose levels (10), it is clear from the literature that TCF7L2 has an important role in glucose metabolism in the liver.

However, important questions remain about the wider impact of TCF7L2-mediated transcription on liver physiology. Given the role of Wnt signaling in regulating diverse pathways in multiple cells and tissues, and it's known role in metabolic zonation of the liver, is TCF7L2 important in determining the overall phenotype of hepatocytes beyond the regulation of carbohydrate metabolism? In an effort to address this question a few studies have examined static TCF7L2 DNA binding across the genome and have shown that TCF7L2 binds to thousands of targets spanning a number of physiological pathways in multiple cell types (9,17–19). However, global DNA binding alone cannot infer function, and it is currently not known which of these TCF7L2 DNA binding sites are functionally significant and regulate target gene expression and, therefore, definitively affect the cellular pathways that impact the hepatocyte phenotype. It also is not clear what mechanisms exist in hepatocytes to regulate TCF7L2 target gene expression. This is particularly relevant in the liver, where cooperation between TCF7L2 and other hepatic transcriptional regulators could significantly enhance the diversity of the pathways regulated by TCF7L2, and allow for fine control of the hepatic phenotype.

In the current study, we employed an integrative genomics study to examine the mechanisms of TCF7L2-target gene regulation in hepatoma cells. Using a combination of temporal RNA-Seq and ChIP-Seq tools in wild-type and TCF7L2-silenced hepatoma cells, we first focused on identifying genes that were directly regulated by TCF7L2 DNA binding. Proximal binding of TCF7L2 was found to preferentially stimulate gene expression in the liver, and only a small fraction of TCF7L2-bound genes were differentially expressed following experimental reductions in TCF7L2 expression. However, these genes included important regulators of multiple pathways critical for liver cell growth, differentiation and cancer, as well as lipid, amino acid and carbohydrate metabolism. We also describe the transcriptional partners of TCF7L2 and, in particular, identify the master-regulator hepatocyte nuclear factor 4-alpha (HNF4α), as a key transcriptional partner of TCF7L2 in the hepatoma cells, particularly in the regulation of metabolic genes. Taken together our data show that direct and indirect regulation of gene expression by TCF7L2 points to a significant role for TCF7L2 in the determination of the hepatic phenotype.

## MATERIALS AND METHODS

### Cell culture, adenovirus infection and siRNA electroporation

Low passage rat H4IIE and human Hep3B cells were purchased from ATCC and used up to passage 30 and 10, respectively. The H4IIE cells were selected for several important reasons. First, it has been shown by many groups that these cells produce glucose in a physiologically relevant manner, a process that is sensitive to insulin and metformin in the physiological range (9,20,21). Second, H4IIE cells have been shown to exhibit highly similar lipid metabolism when compared to primary hepatocytes (21). The human Hep3B cells express wild-type beta-catenin and demonstrate Wnt activity (22). Cells were routinely cultured in low glucose (5 mM) Dulbecco's Modified Eagle Medium (DMEM; H4IIE cells) or Eagle's Minimum Essential Medium (EMEM; Hep3B cells) media supplemented with 10% fetal bovine serum in the absence of antibiotics. Silencing of *Tcf7l2* and/or *Hnf4a* mRNA in H4IIE cells, thereby knocking down TCF7L2 and/or HNF4α protein, was achieved using 100 nM Dharmacon SmartPool siRNA, introduced via Neon electroporation, as described previously (9). Real-time polymerase chain reaction (PCR) were performed using TaqMan^®^ Gene Expression Assays (see Supplementary Methods for catalog numbers), and data were normalized to a panel of three reference targets (*Actb*, *Hmbs* and *B2m*) using qbase+ software (Biogazelle, Zwijnaarde, Belgium). Human wild-type TCF7L2 (Vector Biolabs, cat. #1280) and eGFP control (Vector Biolabs, cat. #1060) were over-expressed in Hep3B cells using adenovirus infected at a multiplicity of infection (MOI) of 50, and harvested 72 h later for western blot and qRT-PCR analysis.

### Time-course RNA-sequencing

Cells were electroporated with TCF7L2 or scramble siRNA in 24-well plates in culture medium for the analysis of RNA-Seq at 3, 6, 9, 12, 15, 18, 48 and 96 h following *Tcf7l2* silencing. The time-course RNA-Seq experiment was performed in duplicate or triplicate with separate electroporations being performed on a different passage of cells on a different date (see data analysis below for more details). RNA was extracted using standard procedures and Illumina sequencing libraries were prepared using the TruSeq RNA Sample Prep Kit V2. More details of the experimental design and sample preparation are provided in the Supplementary Methods.

### Defining the H4IIE-specific transcriptome

The H4IIE-specific transcriptome was defined from a large set of 213.4 million pooled 50 bp single end RNA-seq reads originating from a separate experiment (24 h treatment of TCF7L2 or scramble siRNA, three biological replicates each). See Supplementary Table S1 and Supplementary Methods for detailed read statistics and methodology, respectively.

### Time-course RNA-Seq analysis

The 50 bp single end RNA-seq reads for duplicated or triplicated time points 3, 6, 9, 12, 15, 18, 48 and 96 h after *Tcf7l2* or scramble siRNA treatment were mapped against the H4IIE-specific transcriptome using Tophat version 2.0.4 (with underlying Bowtie version 0.12.8) (23,24), and analyzed for differential expression using Cuffdiff version 2.0.2 (25) (see Supplementary Table S2 for detailed read statistics). Prior to further analysis, the fold changes were recalculated by adding a pseudo count of 0.1 FPKM to the Cuffdiff-reported expression values to eliminate unrealistically high fold changes for genes with very low expression in one of the samples. Per time point, a significant differentially expressed gene (DEG) was required to have a *q*-value < 0.05 when comparing the *Tcf7l2* siRNA treatment against scramble siRNA. Depending on the question, the set of DEGs was further defined separately for each time point, additively over the early (3–18 h) or late (48 and 96 h) time points, or additively over all time points.

### Chromatin immunoprecipitation and ChIP-Seq

Analysis of TCF7L2 and HNF4α genome-wide occupancy was carried out using specific and internally-validated antibodies. For TCF7L2, we used an antibody (clone C48H11, cat. #2569, Cell Signaling Technology, Danvers, MA, USA), that has been previously validated by us for use in western blotting, immunoprecipitations and ChIP-Seq (9) and, more recently, by the Encode Consortium (19). This antibody recognizes a region surrounding Leu330 of the TCF7L2 protein, which is at the 3′ end of exon 9 and is common to all known and theoretical *Tcf7l2* mRNA transcripts. It should be noted, however, that only two major protein isoforms are observed in the H4IIE cells, both of which are detected approximately equally by #2569 on western blots. For HNF4α, a mouse monoclonal antibody (clone H1415, cat. #PP-H1415–00, R&D Systems, Minneapolis, MN, USA) was selected for ChIP-Seq analysis and was validated using the rat apolipoprotein-C-III (Apoc3) gene promoter. A Myc-tag antibody (clone 71D10, cat. #2278, Cell Signaling Technology) was used as a negative control (Supplementary Figure S1). ChIP-Seq was performed on scramble and siRNA-*Tcf7l2* treated cells in duplicate (for TCF7L2) and each sequenced sample (for HNF4α and TCF7L2) represented a pool of two independent immunoprecipitations, each consisting of between 10 and 20 μg of chromatin. Further details on the preparation of ChIP-Seq samples are provided in Supplementary Methods.

### ChIP-Seq analysis

The 50 bp single end ChIP-seq reads for TCF7L2 and HNF4α samples 15 h after *Tcf7l2* or scramble siRNA treatment were mapped against the rn4 reference genome (including the small *Tcf7l2* exon gap fix as described in Supplementary Methods) using Bowtie version 0.12.8 with essential command attributes -n 1 -m 1 -k 1 –trim5 5 -l 36 –best (see Supplementary Table S3 for detailed read statistics). We assessed TCF7L2 binding in the silenced cells primarily to confirm that the reduction in protein levels for TCF7L2 were effectively translated to a global reduction in genome-wide chromatin occupancy during the early time-course period. Each duplicate ChIP-Seq experiment represents an independent siRNA electroporation, performed on separate H4IIE culture passages. The two replicates for each treatment met the ENCODE criteria that 75% of the identified peaks be shared (scramble replicates = 87.1%, siRNA *Tcf7l2* replicates = 94.3% shared, Supplementary Figure S2A) (26), even when we required, stringently, that any two individual peaks overlap only if 50% of the shorter peak was contained within the longer. Peaks were detected using MACS2 callpeak version 2.0.10.20130306 against a common input sample (merged from *Tcf7l2* or scramble siRNA treated cells) with essential command attributes -bw 100 -m 5,50 –keep-dup 1 –qvalue = 0.01 (27). The primary output parameter of statistically significant peaks is the fold enrichment where the enrichment of signal is measured over the ‘noise’ of the input sample. Replicate read sets were subsequently merged, and all downstream analyses were performed on TCF7L2 peaks called from these merged reads.

In order to provide a measure of TCF7L2 or HNF4α binding in *Tcf7l2* and scramble siRNA treated cells for all locations where at least one of the conditions had a peak, the consensus summit set for each transcription factor (TF) was identified as described by us previously (28) (see Supplementary Methods for further details and Supplementary Table S3 for essential peak identification statistics). Final overlap of consensus TCF7L2 and HNF4α peaks was defined using the ‘50% of shorter’ rule described above. Homer software was used for both the *de novo* (version 4.2) and known binding motif searches (version 4.3) (29).

### Integration of DEGs, ChIP-Seq peaks and binding motifs

Custom R-scripts were used to map consensus peaks to the neighborhoods of H4IIE-specific transcripts and genes as well as to integrate the peak-wise Homer motif search results. The increased size, number and/or proximity of binding locations for a given TF around a given DEG has been shown to increase the likelihood that the DEG is a direct target of the TF (30). To calculate such ‘peak proximity scores’, we used the algorithm introduced by Ouyang *et al.* (30) on our TCF7L2 and HNF4α ChIP-seq data in conjunction with the RNA-seq-identified DEGs over the siTcf7l2-treatment time-course (for more detailed description, see Supplementary Methods). To be considered a DEG as a direct target for a given TF, we required that the DEG had a peak proximity score >10 for TCF7L2 or, due to lower overall ChIP-seq signal, >5 for HNF4α.

### Pathway enrichment analysis

Ingenuity pathway analysis (IPA) was used to identify canonical pathways and biological functions that are affected by the siTcf7l2 treatment. Since the early time points of the RNA-seq time course had no or very few DEGs, we chose to perform the analysis for each time point with the top 150 most significantly altered genes from both up- and down-regulated gene sets (total 300 genes), regardless of their actual statistical significance. This allowed the tracking of each affected pathway or function through the entire time course. Custom R-script was used to visualize the enrichment *P*-values as heatmaps.

### Additional statistical tests

Unless otherwise stated, mean ± SD is given, and groups are tested using Welch two-sample *t*-test on log_2_-transformed values after adding a suitable pseudo-count to avoid negative values.

## RESULTS

### Time-course RNA-Seq in TCF7L2-silenced hepatoma cells

**Figure 1.**
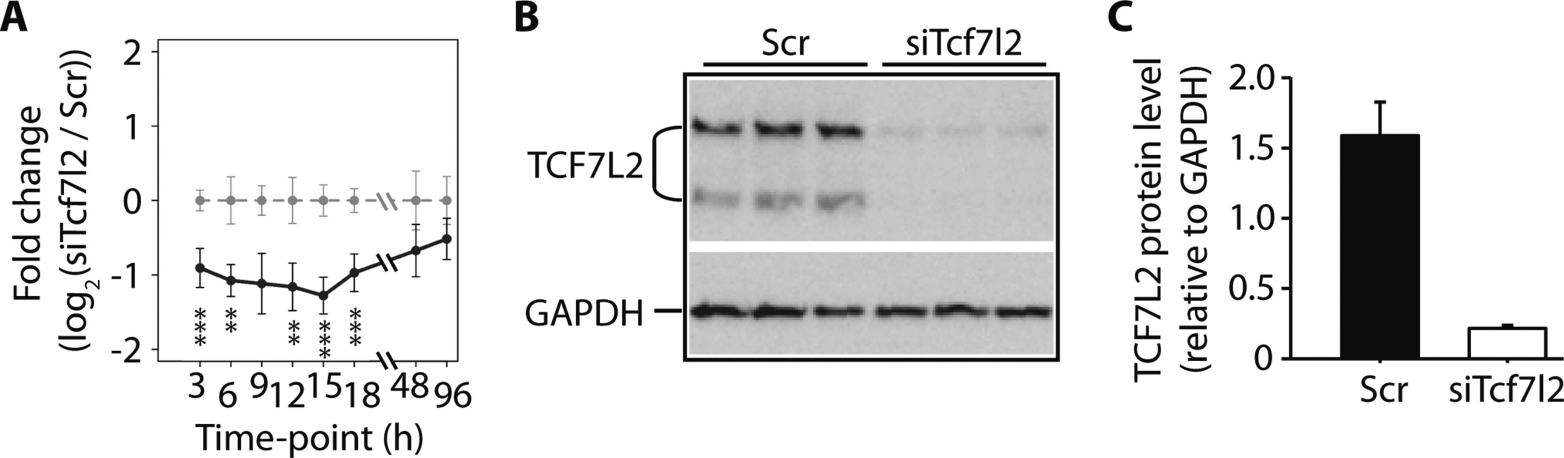
Validation of *Tcf7l2* silencing. H4IIE cells were treated with scrambled (Scr) or *Tcf7l2*-specific (siTcf7l2) siRNA and sampled at various time points for RNA and protein. (**A**) The expression of *Tcf7l2* in Scr and siTcf7l2 treated samples, derived from the RNA-Seq experiment is expressed over the time course as log_2_ of fold change [siTcf7l2/Scr, with 95% confidence intervals for both siTcf7l2 (blue) and Scr (gray)] (*q < 0.05; **q < 0.01; ***q < 0.001). (**B**) Knock-down of TCF7L2 after 24 h siTcf7l2 treatment demonstrated by western blotting. Glyceraldehyde 3-phosphate dehydrogenase (GAPDH) was used as loading control. (**C**) Quantitation of TCF7L2 protein (78 kDa band only) from the western blot image (B), relative to GAPDH and expressed as means ± SEM (*n* = 3 independent experiments).

To begin to understand the mechanisms of target gene regulation by Tcf7l2, we first employed a time-course RNA-Seq approach following transient *Tcf7l2* silencing in hepatoma cells. Our hypothesis was that genes that were rapidly regulated by *Tcf7l2* silencing during our temporal RNA-Seq experiment, and that had a strong proximal TCF7L2 binding site, represented direct TCF7L2 gene targets. As expected, *Tcf7l2* mRNA was rapidly down-regulated at 3 h following siRNA electroporation (−0.9log_2_(FC) versus scramble control, *q* value < 0.001, Figure 1A and Supplementary Tables S4 and S5) and reached maximal knock-down at 15 h (−1.28log_2_(FC), *q* < 1 × 10^−9^), and recovered to ∼70% of the basal levels by 96 h (*q* = 0.485) (Figure 1A). We have previously demonstrated the effect of siRNA on TCF7L2 protein levels 0–96 h after electroporation (9) but, for the purpose of this study, western blots were performed 24 h after *Tcf7l2* silencing to confirm successful knock-down at protein level (Figure 1B and C). TCF7L2 protein knock-down was also confirmed 15 h post-electroporation in latter ChIP-Seq studies (Supplementary Figure S2; see below).

**Figure 2.**
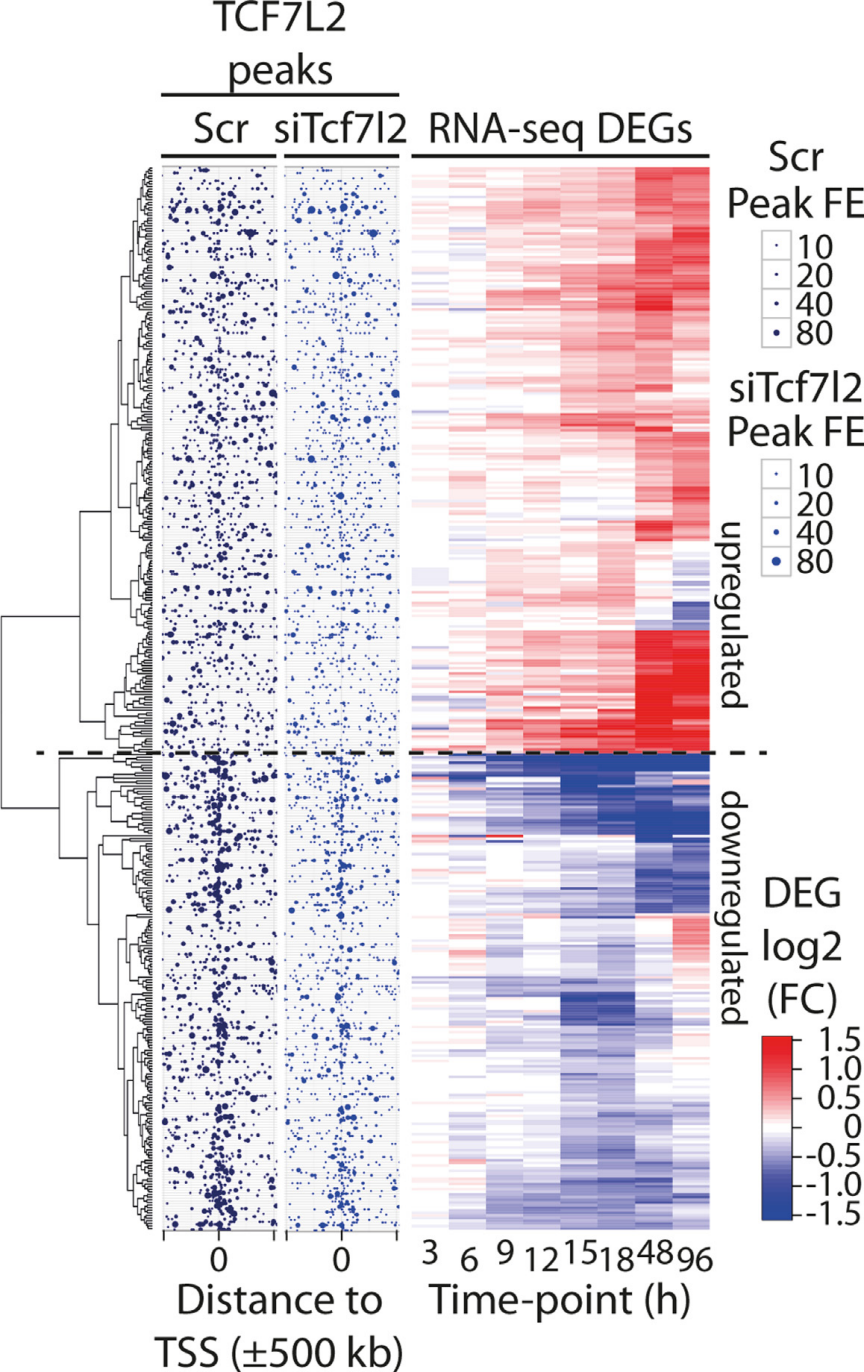
Temporal changes in gene expression and TCF7L2 chromatin occupancy upon *Tcf7l2* silencing. H4IIE cells were treated with scrambled (Scr) or *Tcf7l2*-specific (siTcf7l2) siRNA and sampled at various time points for ChIP-seq (15 h) and RNA-seq (3–96 h) analysis. The heat map on the right displays hierarchically clustered log_2_ fold changes (FC, siTcf7l2/Scr) for all 406 differentially expressed genes (DEGs) that were significantly altered at any one of the time points indicated below the heat map. Red color scale indicates up-regulation and blue color scale down-regulation (for visualization, the extremes are saturated at 1.5 or −1.5, respectively). The respective dendrogram for the gene-axis is shown at the left. The two mid panels display the locations of all high-confidence (FDR < 0.01) TCF7L2 ChIP-seq peaks within ±500 kb around the transcriptional start site (TSS) of the respective DEG, ordered to match the heat map. The point size reflects the ChIP-seq peak fold enrichment (FE) over Input sample as indicated. Note the increased density of TCF7L2 peaks near the TSSs of the down-regulated DEGs (below the horizontal dashed line), and the decrease in peak size due to the siTcf7l2 treatment.

As shown in Table 1, there was a time-dependent increase in the number of DEGs following *Tcf7l2* silencing. A total of 406 genes were identified as a DEG in at least one time point following *Tcf7l2* silencing. Only three DEGs were identified prior to 15 h after *Tcf7l2* silencing, and 97, 175, 38 and 92 unique DEGs were identified at 15, 18, 48 and 96 h, respectively, after electroporation (Table 1 and Figure 2). Inspection of the RNA-Seq data revealed the presence of two distinct sets of DEGs. Of the 276 DEGs that changed early in the RNA-Seq time-course (3–18 h), only 78 remained among the 208 late DEGs (48 or 96 h), suggesting a temporal shift between early, more directly-regulated TCF7L2 targets, and latent changes in gene expression as a result of indirect regulation by TCF7L2.

**Table 1.** Differentially expressed genes at various time points, or sets thereof, following *Tcf7l2* silencing in H4IIE hepatoma cells

		DEGs		
Phase	Time (h)	New per time point	Cumulative	Total per phase
Early	3	3	3	276
	6	0	3	
	9	0	3	
	12	1	4	
	15	97	101	
	18	175	276	
Late	48	38	314	208
	96	92	406	
Total (DEGs)		406	406	406

### Multiple pathways are regulated by TCF7L2-silencing in hepatoma cells

To understand the biological functions and pathways regulated by TCF7L2, we first performed IPA on the 406 DEGs from the complete time-course RNA-Seq experiment. From the top 20 BioFunctions, the major themes of lipid metabolism and cell growth, differentiation and cancer were highly enriched (Supplementary Table S6). Genes including E-cadherin (*Cdh1*, up), amphiregulin (*Areg*, down), c-Met/hepatocyte growth factor receptor (*Met*, down) and c-Myc (*Myc*, down) were notable examples of genes involved in cell growth, differentiation and cancer that were significantly regulated by *Tcf7l2* silencing. The enrichment of multiple lipid metabolism and transport pathways in our full IPA analysis was the result of marked changes in the expression of genes important for lipid uptake, synthesis, storage and transport. Liver-specific fatty-acid binding protein-1 (*Fabp1*), apolipoprotein B (*Apob*), stearoyl-CoA desaturase-1 (*Scd1*), diglyceride acyltransferase-2 (*Dgat2*), microsomal triglyceride transfer protein (*Mttp*) and the ATP-binding cassette sub-family G member 8 (*Abcg8*) were all up-regulated by *Tcf7l2* silencing.

**Figure 3.**
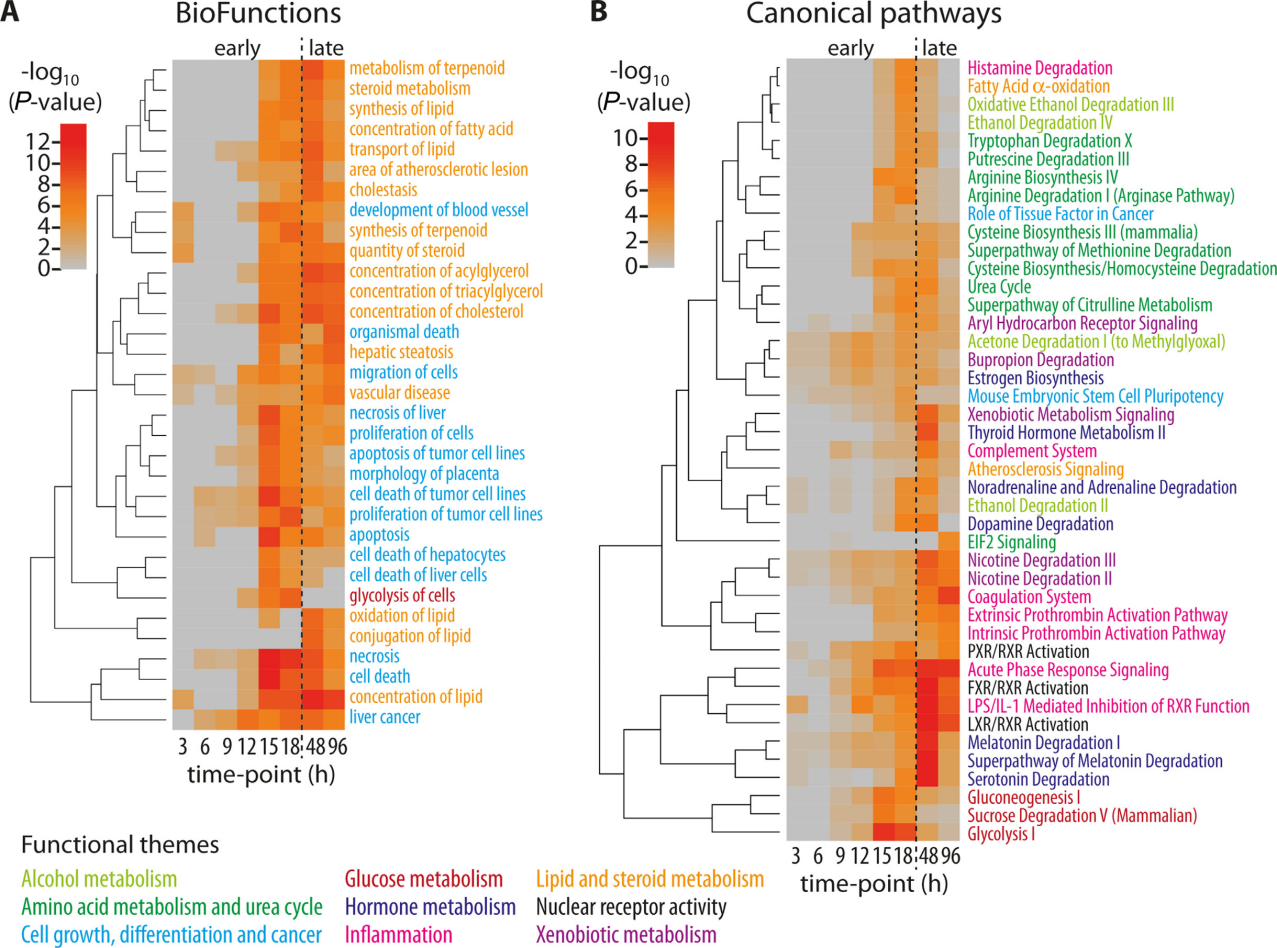
Pathway enrichment of genes with altered expression along the *Tcf7l2* silencing time course. For each time point, the ingenuity pathway analysis (IPA) was performed on the top 150 up- and 150 down-regulated genes, regardless of their actual statistical significance. The –log_10_-transformed IPA *P*-values are displayed as hierarchically clustered heat maps for top enriched. (**A**) The BioFunction (*P* < 10^−7^ at any time point) and (**B**) the canonical pathway (*P* < 0.001 at any time point) collections, as indicated. In both A and B, the function or pathway names are color coded for membership in the indicated broader themes, and the vertical dashed line separates the early (3–18 h) and late (48 and 96 h) phases of the time course.

To exploit the temporal aspect of our RNA-seq data, we next performed IPA separately at each point along our time course using 300 genes (150 each from up- and down-regulated genes) that were the most significantly affected following siRNA treatment (irrespective of *q*-value). As shown in Figure 3, the top enriched BioFunctions (Figure 3A) and canonical pathways (Figure 3B) represented nine broad categories. Those BioFunctions that belonged to the category of lipid and steroid metabolism appeared also in this analysis, but their enrichment typically peaked at the later time points (Figure 3A). In contrast, BioFunctions enriched early belonged primarily to the broader category of cell growth, differentiation and cancer, functions strongly associated with Wnt signaling in multiple tissues (Figure 3A). Other canonical pathways belonged to the broader categories of alcohol, hormone, or xenobiotic metabolism, inflammation, or nuclear receptor activity, and also were enriched late in the time course.

**Figure 4.**
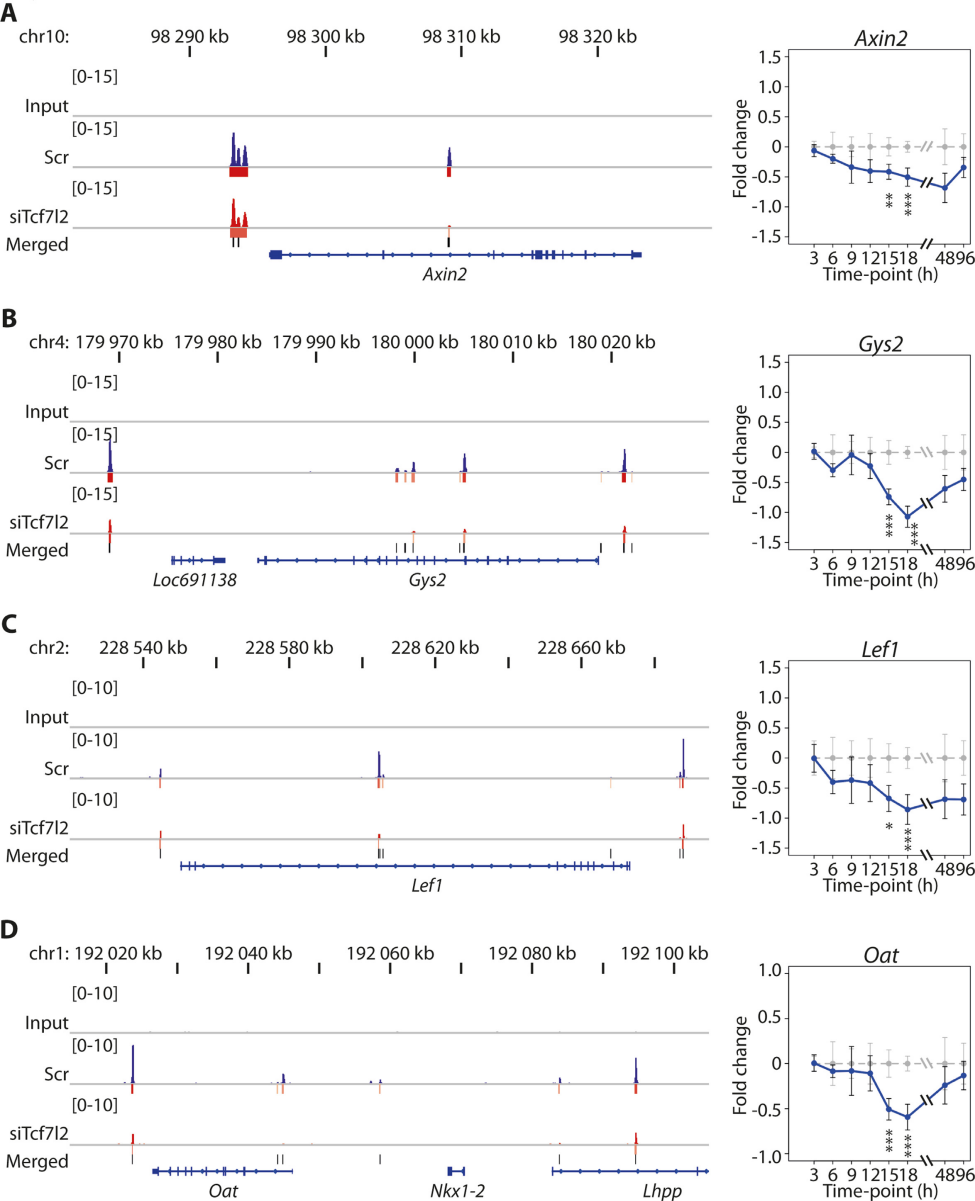
TCF7L2 binds to and regulates multiple Wnt/β–catenin pathway and metabolic genes in hepatoma cells. On the left, ChIP-seq-derived occupancy of TCF7L2, and on the right, the change in gene expression following *Tcf7l2* silencing for (**A**) *Axin2*, (**B**) *Gys2*, (**C**) *Lef1* and (**D**) *Oat*. On the left panels, normalized TCF7L2 ChIP-seq read accumulation tracks are displayed for Input, Scr and siTcf7l2 samples collected at 15 h siRNA treatment. High-confidence (FDR < 0.01) ChIP-seq peaks are shown under the Scr and siTcf7l2 tracks with bars whose coloring (from pale yellow to red) indicates the peak size (fold enrichment over Input). Merged peaks are displayed as black bars above the gene body track in blue. On the right panels, the RNA-seq-derived gene expression over the time course is expressed as log_2_ of fold change (siTcf7l2/Scr, with 95% confidence intervals for both siTcf7l2 (blue) and Scr (gray)). Statistical significance (Cuffdiff *q*-value) at a given time point is indicated by stars (*q < 0.05; **q < 0.01; ***q < 0.001).

Canonical amino acid metabolism pathways—notably arginine, cysteine, tryptophan, methionine and citrulline—and urea metabolism, were also highly enriched in our temporal IPA analysis (Figure 3B). For example, ornithine aminotransferase (OAT, encoded by *Oat*)—which regulates ornithine synthesis from citrulline—was down-regulated by Tcf7l2 silencing, and was a direct target of TCF7L2, as discussed below (Figure 4). The genes of such key enzymes of the urea cycle as *Ass1*, *Asl*, Arginase (*Arg1*) and carbamyl phosphate synthetase 1 (*Cps1*) were up-regulated by *Tcf7l2* silencing. The enrichment for the majority of these pathways peaked either at 18 or 48 h time point.

Gluconeogenesis and glycolysis pathways were enriched early at 15–18 h post-silencing (Figure 3), which was due to the large number of rate-limiting glycolytic and gluconeogenic genes that were differentially regulated by *Tcf7l2* silencing, including *Pfkl* (6-phosphofructokinase, down), *Slc2a1* (facilitated glucose transporter, member 1, down), *Gys2* (glycogen synthase 2, down), *Aldob* (fructose-bisphosphate aldolase B, up), *Eno2* (enolase 2, down), *G6pc* (glucose 6-phosphatase, up) and *Fbp2* (fructose-1, 6-bisposphatase 2, up) (Supplementary Table S4 and S5). Genes associated with the uptake, utilization, oxidation and storage of external glucose were down-regulated by *Tcf7l2* silencing, while genes essential for the production of glucose were up-regulated, consistent with the hypothesis that TCF7L2 is an important regulator of hepatic glucose metabolism. Collectively, these pathway data demonstrate that TCF7L2 has a diverse metabolic and non-metabolic role in liver cells and that TCF7L2 levels are important for the expression of a number of critical metabolic pathway mediators.

### Combined ChIP-Seq and RNA-Seq reveals genes directly regulated by TCF7L2

Our temporal RNA-Seq experiments suggested the presence of an early and late set of DEGs, and we were interested in whether the early DEGs were more likely to be direct targets of TCF7L2. To gain further insight into the direct regulation of gene expression by TCF7L2, we investigated genome-wide chromatin occupancy of TCF7L2 in the control and *Tcf7l2*-silenced cells 15 h after TCF7L2 knock-down. There was an approximate 80% reduction in the number of observed peaks in siTcf7l2 cells 15 h after siRNA electroporation (8446 versus 1573 total peaks with 97% overlap; Supplementary Figure S2A, and Supplementary Table S7). On average, TCF7L2 ChIP-Seq peak enrichment also was reduced by 60.4% (*P* < 2.2 × 10^−16^) across the genome, as shown globally in Supplementary Figure S2B and at selected target genes, including *Tcf7l2* itself, in Supplementary Figures S2C, S3 and S4 (full GEO datasets: GSE53862). The reduced enrichment was not due to lower number of sequenced or mapped reads (Supplementary Table S3), and was confirmed using ChIP qRT-PCR at the *Axin2* gene (Supplementary Figure S2D).

Superimposing TCF7L2 ChIP-Seq and siTcf7l2 RNA-Seq data revealed that 149 (54.0%) of the early DEGs contained strong proximal TCF7L2 binding sites (TCF7L2 peak proximity score > 10), compared to only 51 (39.2%) of the unique late DEGs, indicating that a greater proportion of the early DEGs are likely directly regulated by TCF7L2 (Supplementary Tables S4 and S5). This is supported by the temporal nature of the early gene expression changes; *Tcf7l2* mRNA was significantly reduced at 3 h and global TCF7L2 DNA binding also was reduced at 15 h by ∼60%, yet up to 15 h post-*Tcf7l2* silencing only three other genes were found to be differentially expressed. The number of DEGs increased markedly at 18 h, however, and this latency period between the reduced *Tcf7l2* expression and downstream gene target effects suggests a mechanistic link between *Tcf7l2* silencing and downstream gene regulation. The relationship between the TCF7L2 binding and temporal changes in nearby gene expression genome-wide is summarized in Figure 2.

**Figure 5.**
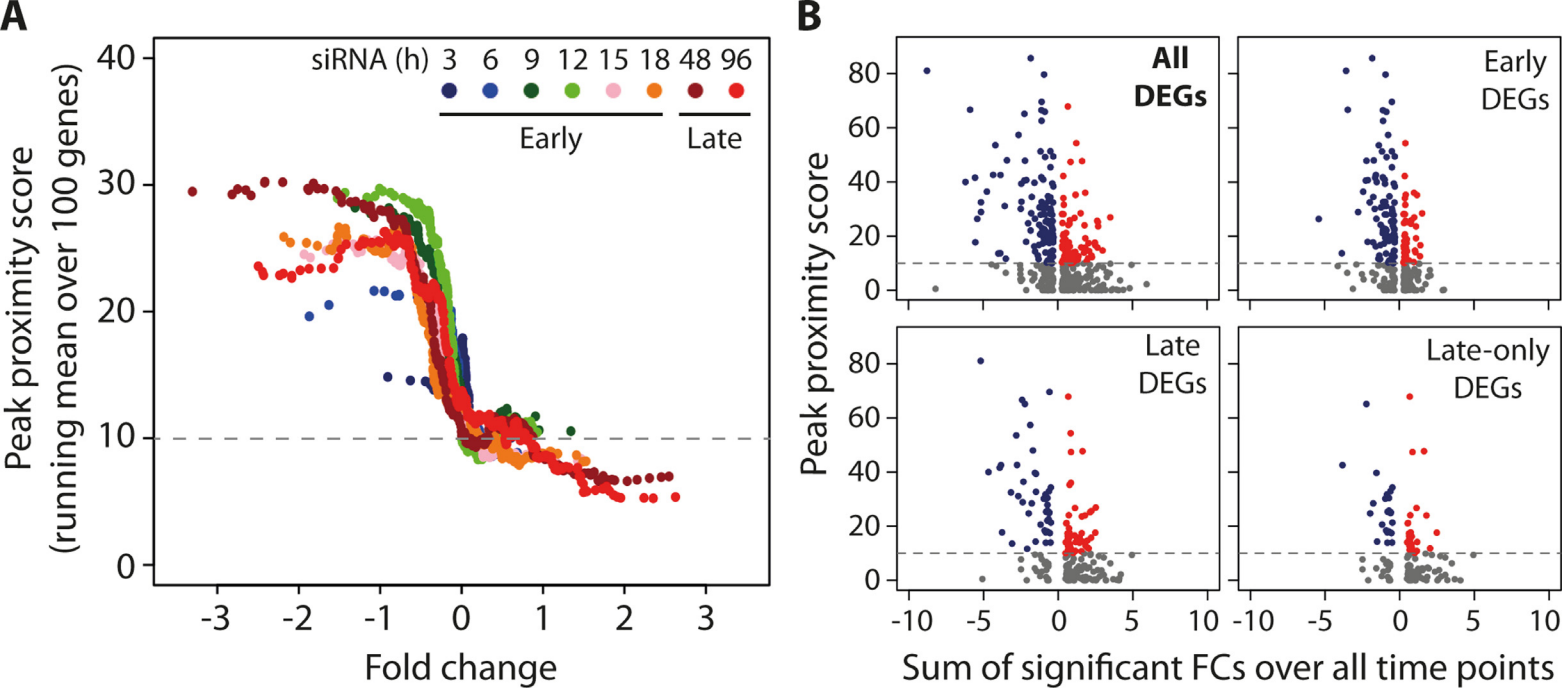
TCF7L2 preferentially binds near genes down-regulated by the siTcf7l2 treatment. TCF7L2 peak proximity scores for each of the full set of 406 differentially expressed genes (DEGs) were calculated and plotted together with DEG log_2_ of fold change (FC, siTcf7l2/Scr) values. (**A**) For each time point, the genes were ordered according to their FC and plotted against the peak proximity score running means over each consecutive set of 100 genes. Time points are color coded as indicated. (**B**) Gene expression changes over the time course were summarized as cumulative FC values over the time points at which the gene was significantly affected, considering either all time points (‘All DEGs’) or the indicated subset, and plotted against the respective TCF7L2 peak proximity score. ‘Late’ genes include all genes that are DEGs at either of the late time points, irrespective of their status during the ‘Early’ time points. ‘Late-only’ are those that are DEGs exclusively at either of the late time points. Blue dots indicate negative and red positive sum of significant FCs. In both (A) and (B), the horizontal dashed line at peak proximity score 10 indicates the chosen cut-off for defining a gene likely to be a direct target of TCF7L2.

Of note, the TCF7L2 peaks were much more frequent around genes that were down-regulated by *Tcf7l2* silencing compared to those that were up-regulated (lower versus upper half of Figure 2), which was confirmed when the running means of peak proximity scores were plotted against the sorted DEG fold-changes (FCs) at each time point (Figure 5A); down-regulated DEGs clearly had significantly higher peak proximity scores for TCF7L2 compared to up-regulated DEGs (Figure 5A). Figure 5A also highlights the temporal relationship between proximal TCF7L2 binding and the down-regulation of target genes, as the running mean peak proximity score increases through the early phase of the time-course and saturates by 12 h. This temporal relationship also was evident when average peak proximity scores for directly targeted down- and up-regulated DEGs were compared within the early and late-only DEG sets (Figure 5B). The difference is greater and more significant for early DEGs (early: 31.1 ± 17.2 versus 19.4 ± 9.4, *P* < 1 × 10^−6^; late-only DEGs: 26.8 ± 11.5 versus 20.0 ± 13.2, *P* < 0.006).

As outlined above, our IPA analysis indicated that early DEGs were more likely to be related to Wnt pathway functions, and we have highlighted *Axin2* and *Lef1* genes as archetypal direct TCF7L2 target genes in Figure 4. The IPA analysis also indicated that many metabolic pathways were well represented by our set of DEGs, including amino acid, urea, lipid and glucose metabolism, and for the first time, our data provide evidence that many, but not all, genes associated with these pathways are directly regulated by TCF7L2 proximal DNA binding activity. As an example of this, the *Gys2* and *Oat* genes were down-regulated in a time-dependent manner in hepatoma cells following *Tcf7l2*-silencing, and each displayed clear reductions in proximal TCF7L2 binding at 15 h (Figure 4). Further examples of potential direct TCF7L2 metabolic genes are highlighted in Supplementary Figure S3 and full datasets are available online (GEO: GSE53862). Together these data highlight that TCF7L2 DNA occupancy can directly regulate the expression of multiple cellular and metabolic genes in hepatoma cells, many of which have not previously been shown to be directly regulated by TCF7L2.

### TCF7L2 regulates multiple transcription factors in hepatoma cells

**Figure 6.**
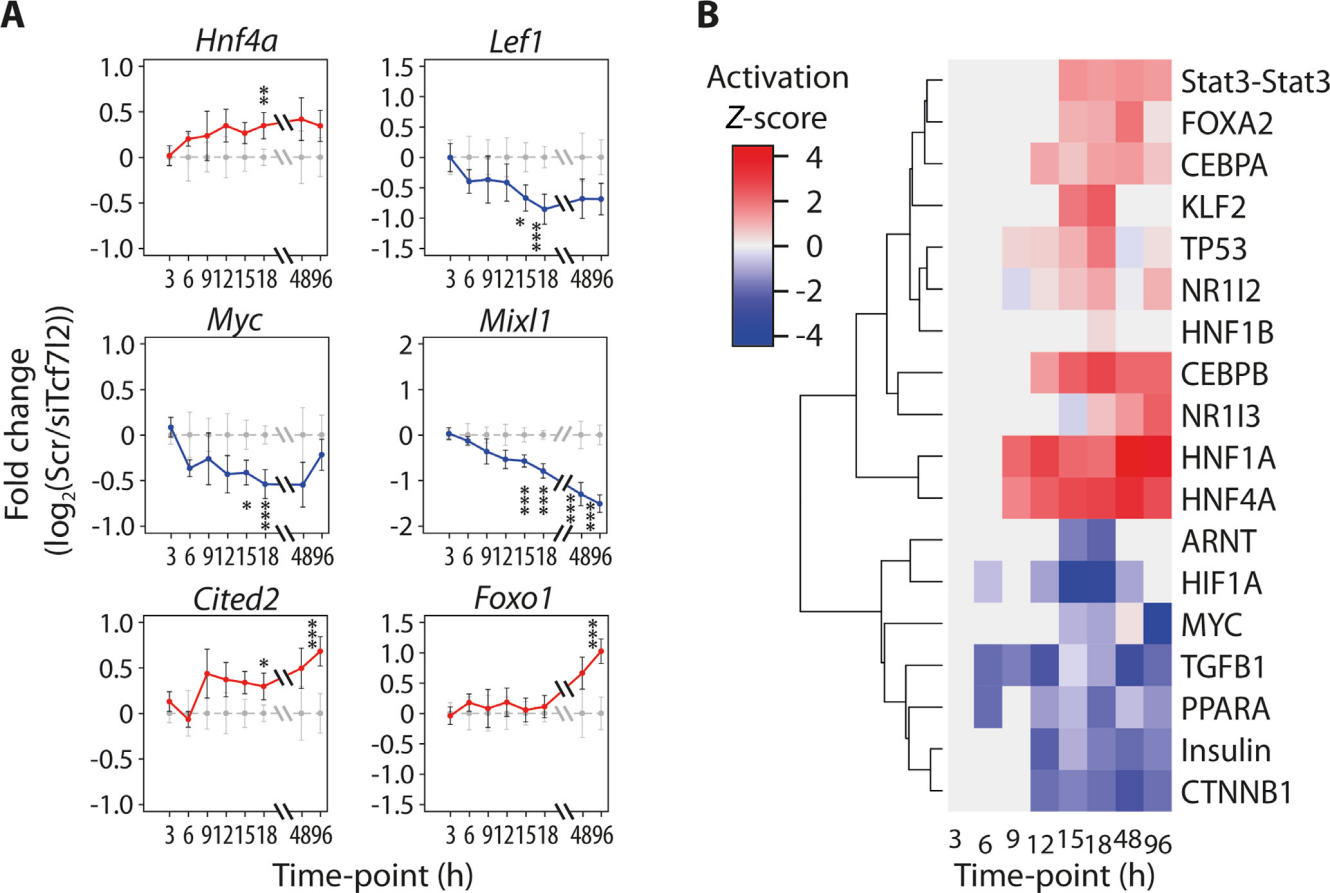
Silencing of *Tcf7l2* alters the gene expression of hepatic transcription factors and their target genes. (**A**) The change in expression, as measured by RNA-seq, is displayed as log_2_ of fold change (siTcf7l2/Scr, with 95% confidence intervals for both siTcf7l2 (blue for down-regulation and red for up-regulation) and Scr (gray)) along the full time course for *Hnf4a*, *Lef1*, *Myc*, *Mixl1*, *Cited2* and *Foxo1*, as indicated. Statistical significance (Cuffdiff *q*-value) at a given time point is indicated by stars (*q < 0.05; **q < 0.01; ***q < 0.001). (**B**) For each time point, ingenuity upstream regulator analysis was performed on the top 150 up- and 150 down-regulated genes, regardless of their actual statistical significance. The Activation *Z*-scores are displayed as hierarchically clustered heat map for top enriched regulator (*P* < 10^−9^) at any time point. Red suggests up-regulation and blue down-regulation by the regulator, and gray indicates missing values.

Between 45 and 60% of all DEGs did not have proximal TCF7L2 binding peaks, and this led us to examine what factors may be regulating the DEGs that did not possess TCF7L2 binding peaks. A lack of proximal TCF7L2 binding was more evident for late-appearing DEGs (60%) and genes that were up-regulated by *Tcf7l2* silencing (Figures 2 and 5). Interestingly, we found several transcription factors amongst our RNA-Seq data that were differentially regulated by *Tcf7l2* silencing (Figure 6A and Supplementary Figure S4), which included important transcriptional regulators of liver cell development, oncogenesis and metabolism. Of particular interest, *Hnf4a* (first significant time point = 18 h, log_2_ fold-change 0.35, *q* value = 0.007), *Lef1* (15 h, −0.67 FC, *q* = 0.017), *Myc* (15 h, −0.41 FC, *q* = 0.025), *Mixl1* (15 h, −0.57 FC, *q* < 0.001), *Cited2* (18 h, 0.3 FC, *q* = 0.047) and *Foxo1* (96 h, 1.0 FC, *q* = 8 × 10^−6^) were all affected by TCF7L2 knock-down. We examined whether these factors may be directly regulated by TCF7L2. TCF7L2 binding sites were identified internally and proximal to the *Hnf4a* gene, and were reduced following *Tcf7l2* silencing. Both *Mixl1* and *Lef1* contained proximal TCF7L2 ChIP-Seq peaks, which also were reduced 15 h after silencing *Tcf7l2* (Supplementary Figure S4A and Figure 4C). These data suggest that *Tcf7l2*-silencing may directly regulate the expression of many alternate transcription factors that may affect downstream gene targets that are not bound by TCF7L2. However, *Foxo1* (90 kb), *Myc* (300 kb), and *Cited2* (75 kb) contained no proximal TCF7L2 binding peaks and, thus, may be regulated by a transcription factor(s) other than TCF7L2 (Supplementary Figure S4B–D). To gain additional insight into the transcriptional processes that might regulate the DEGs in our study, we performed an upstream regulators analysis in IPA. The activation *Z*-scores for top enriched upstream regulators further highlight the roles of activated HNF4α behind the up-regulation and inactivated Wnt/β–catenin signaling (CTNNB1 in the figure) behind the down-regulation of genes whose expression is altered upon silencing of *Tcf7l2* (Figure 6B). The down-regulation of insulin signaling and PPARα transcription in this analysis reflects the increased expression of gluconeogenesis and lipid synthesis and trafficking genes in the hepatoma cells following *Tcf7l2* silencing, as both a negative regulators of these pathways.

### Co-occurring binding motifs

**Figure 7.**
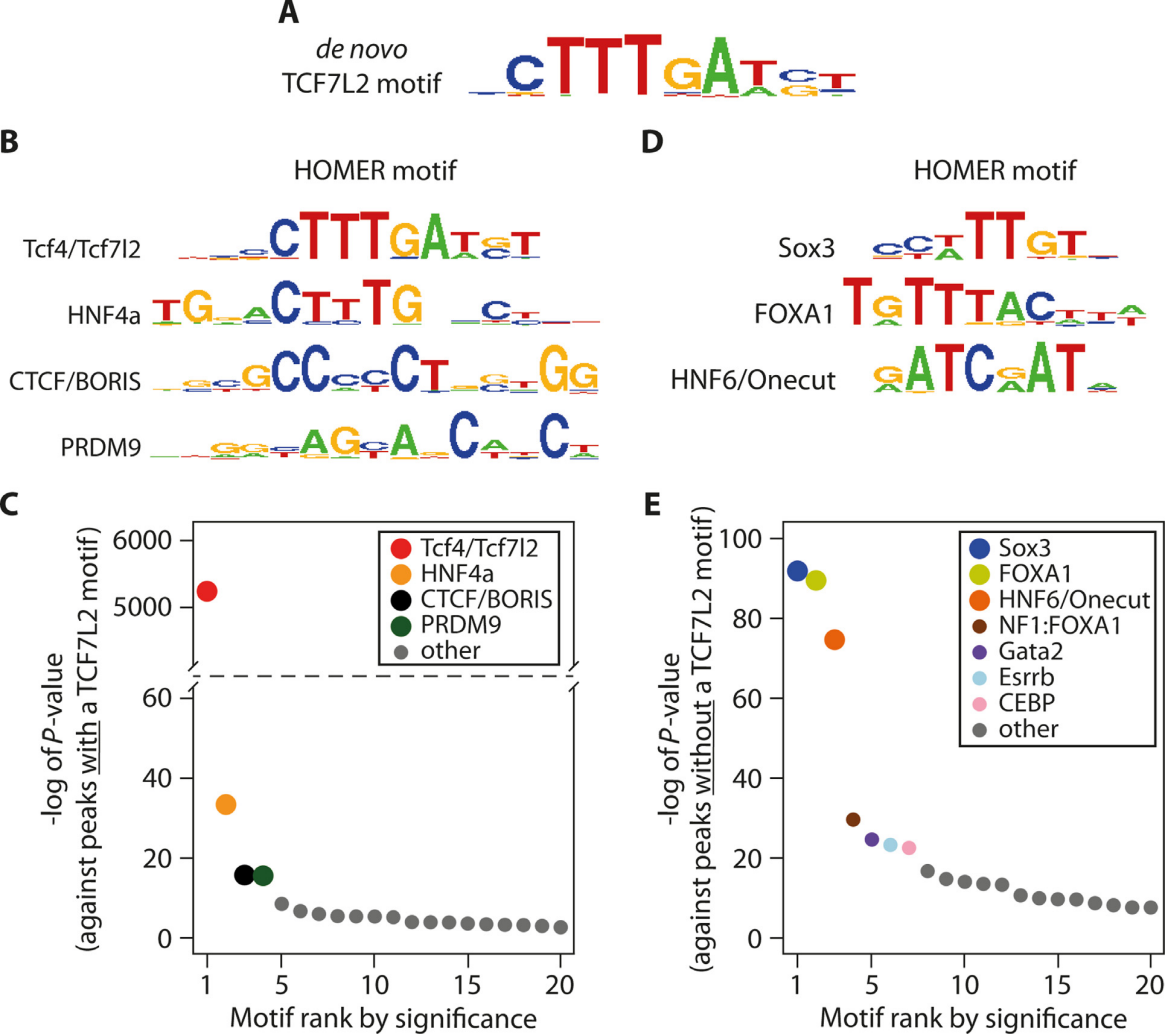
Motif enrichment at TCF7L2 binding sites depends on the presence of a TCF7L2 binding sequence. (**A**) The best *de novo* binding motif identified within ±100 bp of TCF7L2 ChIP-seq peak summits, and known motifs enriched in TCF7L2 peaks (±100 bp of peak summit) with (**B** and **C**) or without (**D** and **E**) the *de novo* TCF7L2 motif. In (C) and (E), the –log of enrichment *P*-values are plotted in ranked order for the top 20 most significantly enriched motifs, out of which a select few most significant are named as indicated and displayed as sequence logos in (B) and (D), respectively. Motif analyses were performed using HOMER.

Many target genes are co-regulated by multiple transcription factors in a cell-type specific manner, and to further investigate the transcriptional partners of TCF7L2, we subjected TCF7L2 ChIP-Seq peaks to binding motif analysis. Of the 8533 total TCF7L2 binding locations (combined Scr and siTcf7l2 peaks), 3706 (43.4%) had a significant (HOMER score > 8.005008) H4IIE *de novo* TCF7L2 motif within ± 100 bp of the summit (Figure 7A and B). However, in peaks with the *de novo* TCF7L2 motif, the most significant co-enriched motifs were for HNF4α, PRDM9 and BORIS/CTCF (Figure 7B and C). In TCF7L2 ChIP-Seq peaks that did not contain the conserved TCF7L2 motif, Sox3, FOXA1 and HNF6/Onecut motifs were significantly enriched (Figure 7D and E). Although sequence similarities between these motifs were noted, there were distinct differences, including the preference for a T nucleotide in the seventh position of the Sox3 motif and alternate nucleotides surrounding the core TTT sequence in the FOXA1 motif (Figure 7A and B). With the RNA-Seq data, this motif analysis supports an interaction between TCF7L2 and a number of alternative hepatic transcriptional regulators, and further highlights a potential interaction between TCF7L2 and HNF4α to regulate target genes.

### HNF4α and TCF7L2 cooperate in their function in hepatoma cells

Because HNF4α is a known master regulator of metabolic gene expression in the liver and is strongly associated with MODY forms of diabetes, we focused in more detail on the relationship between TCF7L2 and HNF4α at the molecular and genomic level. Consistent with the mRNA data above, *Tcf7l2* silencing led to an increase in HNF4α protein levels 24 and 48 h after electroporation in hepatoma cells (Supplementary Figure S5). In human Hep3B cells that express wild-type β-catenin, over-expression of human TCF7L2 also reduced HNF4α mRNA and protein levels significantly (Supplementary Figure S6). Binding peaks for TCF7L2 were observed in intron 3 and ∼13 kb downstream of the *Hnf4a* gene, and HNF4α also bound multiple sites around its own gene, suggesting the existence of a positive feedback loop on *Hnf4α* gene expression (Supplementary Figure S7A).

Chromatin occupancy of HNF4α increased following *Tcf7l2* silencing by ∼50% (2373 versus 3563 peaks in scramble and siTcf7l2 cells, respectively) (Supplementary Table S8). In contrast to TCF7L2, however, HNF4α DNA binding associated strongly with genes that were up-regulated after siTcf7l2 treatment (Supplementary Figure S8A). The difference in HNF4α peak proximity scores between down- and up-regulated genes applied to both early- and late-appearing DEGs (early: 11.9 ± 6.3 versus 15.8 ± 10.1, *P* < 0.02; late-only DEGs: 9.1 ± 5.1 versus 13.2 ± 8.7, *P* < 0.03) (see also Supplementary Figure S8B). The specificity of the HNF4α chromatin binding was confirmed by motif analysis demonstrating enrichment of the HNF4α consensus site (TGRACTTTGVMCYYTG, Supplementary Figure S7B) in both control and silenced cells. As mentioned above, it is noteworthy that the core sequence in the HNF4α motif (ACTTTG) is highly similar to that in the conserved TCF7L2 motif (Figure 7A), although outside that core sequence, there are a number of important differences like the position immediately 3′ of the core where TCF7L2 has a near absolute requirement for A whereas HNF4α accepts any nucleotide.

**Figure 8.**
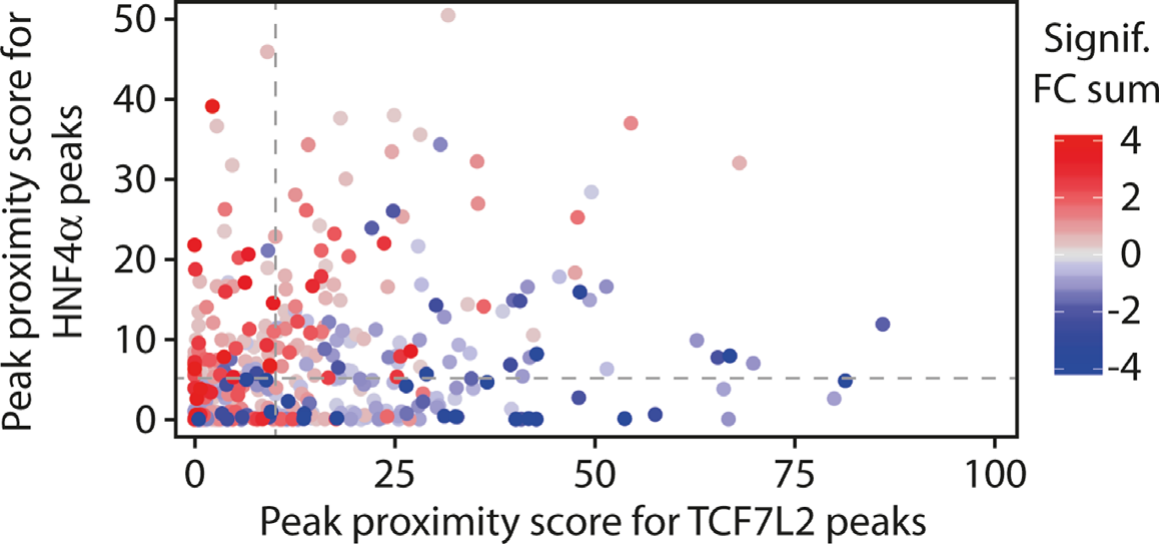
Differential association of TCF7L2 and HNF4α binding near genes down- or up-regulated by siTcf7l2. The scatterplot shows the TCF7L2 (*x*-axis) and HNF4α (*y*-axis) peak proximity scores for each gene in the full set of 406 differentially expressed genes. The points are colored as indicated according to the cumulative FC values summed over those time points at which the gene was statistically significantly affected. The dashed gray lines indicate the chosen cut-offs for defining the genes likely to be direct targets of TCF7L2 (those right of the vertical line) or HNF4α (those above the horizontal line).

Of the total combined 11 793 binding sites for TCF7L2 and HNF4α, 1050 were shared between TCF7L2 and HNF4α (∼9%). This is most likely a conservative estimate of TCF7L2 and HNF4α binding overlap since we reserved classifying a binding site as shared to TCF7L2 peaks that contained at least 50% of an HNF4α peak (or vice versa), and peaks were narrow (∼±100 bp). The ChIP-seq read accumulations around the combined 11 793 peaks are visualized in Supplementary Figure S9. Integrating TCF7L2 and HNF4α peak proximity scores for the 406 DEGs confirmed that genes targeted exclusively by TCF7L2 (peak proximity score for TCF7L2 > 10 and for HNF4α ≤ 5) were preferentially down-regulated upon siTcf7l2 treatment (63 DEGs down, 21 up), whereas HNF4α-exclusive targets (peak proximity score for TCF7L2 ≤ 10 and for HNF4α > 5) were primarily up-regulated (9 DEGs down, 65 up) (Figure 8). Interestingly, ∼29% (116) of all DEGs were targeted by both TCF7L2 and HNF4α (peak proximity score for TCF7L2 > 10 and for HNF4α > 5), irrespective of whether the individual binding locations were shared by the two TFs.

Interestingly many metabolic genes regulated and occupied by TCF7L2 binding also were co-bound by HNF4α (Supplementary Figure S10A–D). This was especially notable at some lipid (*Dgat2*) and gluconeogenic gene (*Fbp1*, *Fbp2*) loci, and HNF4α binding sites were also apparent at several transcription factor genes mentioned above (*Foxo1*, *Cited2*) (Supplementary Figure S10E–H). However, binding of HNF4α at Wnt/β-catenin target genes, including *Tcf7l2*, was minimal (Supplementary Figure S11), i.e. they are regulated mainly by TCF7L2.

### Double-silencing reveals metabolic genes directly regulated by HNF4α

**Figure 9.**
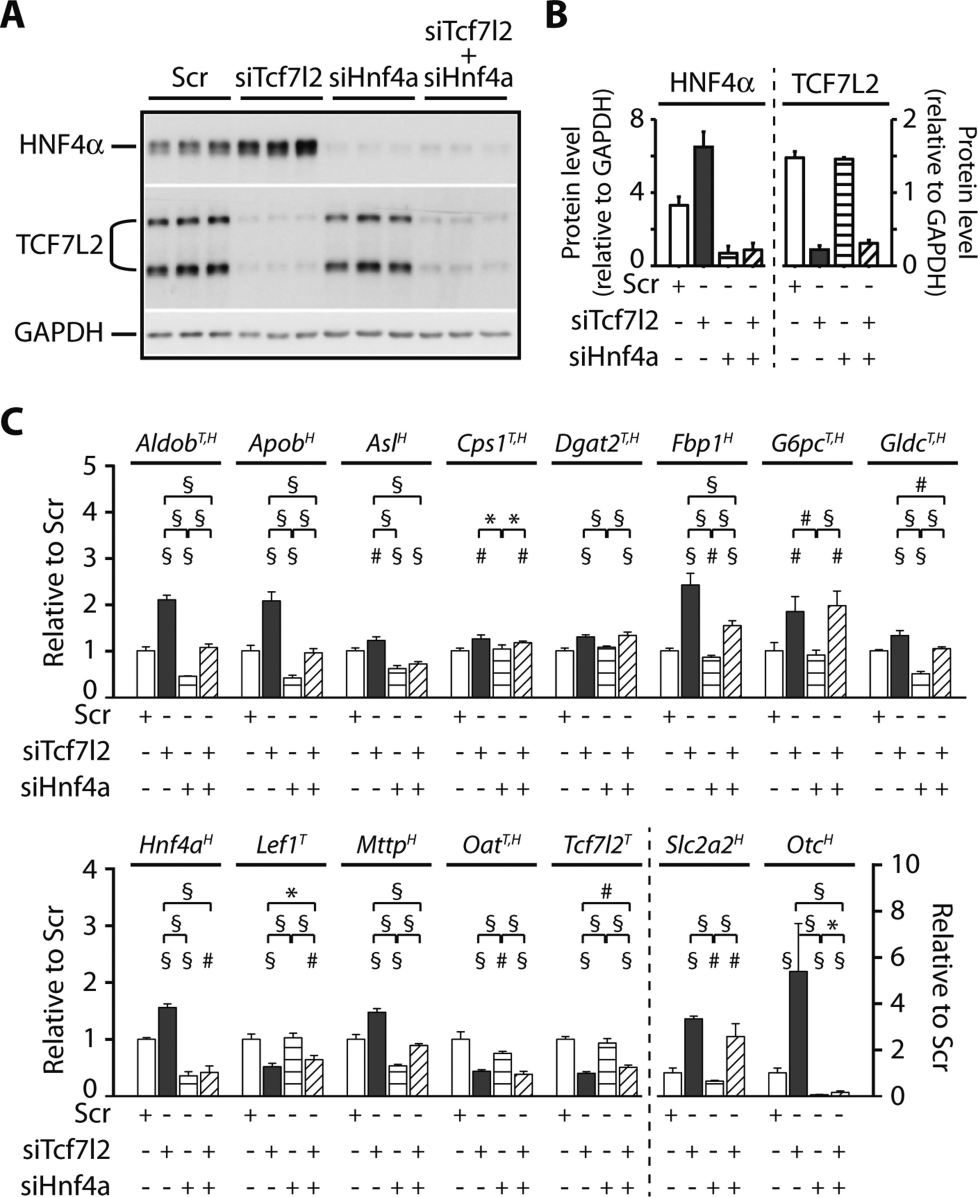
Changes in select metabolic genes upon co-silencing of TCF7L2 and HNF4α. H4IIE cells were treated with scrambled (Scr), *Tcf7l2*-specific (siTcf7l2), *Hnf4a*-specific (siHnf4a) siRNA, or both siTcf7l2 and siHnf4a, and sampled at 48 h for protein. (**A**) Successful knock-down of HNF4α and TCF7L2 protein after the indicated siRNA treatments demonstrated by western blotting. The western blot shown represents a single silencing experiment with three technical replicates for each treatment, and this experiment was repeated three times for quantification. HNF4α and TCF7L2 were probed on separate blots and a GAPDH loading control was performed for each, but for simplicity, only one GAPDH blot is shown. (**B**) Quantitation of HNF4α and TCF7L2 protein from the western blot experiments (A), relative to each GAPDH and expressed as means ± SEM (*n* = 3 independent experiments with three technical replicates for each treatment). (**C**) Relative expression of select metabolic and TF genes after the indicated siRNA treatments, measured by quantitative RT-PCR, normalized to three housekeeping genes and expressed relative to Scr treatment (mean ± SD, *n* = 4 independent experiments, each experiment with three technical replicates per treatment). Superscript letters after gene names indicate the presence of proximal TCF7L2 and/or HNF4α peaks based on the peak proximity score cutoffs (T, TCF7L2 > 10; H, HNF4α > 5). For Student's *t*-tests between Scr and the other treatments, the values were log_2_-transformed and *P*-values corrected for multiple testing using FDR. For clarity, test results between Scr and other groups are indicated without a connecting line, and only by a marker above the non-Scr group bar in each comparison. For all comparisons, *P < 0.05; ^#^*P* < 0.01; ^§^*P* < 0.001.

Finally to test whether HNF4α was required for the full effect of *Tcf7l2* silencing on gene expression in the hepatoma cells, we analyzed the effect of *Hnf4a* silencing with and without concomitant *Tcf7l2* silencing on a subset of our DEGs. For this experiment, we chose, in addition to *Tcf7l2* and *Hnf4a*, 13 genes across a range of pathways that were enriched in our IPA analysis, including amino acid and urea metabolism, lipid metabolism and transport, cell growth and differentiation and glucose metabolism. These 13 genes included *Otc*, which was not quantified in our RNAseq data analysis due to its very low expression levels, but given the changes in other urea metabolism genes in our dataset, we wished to measure the expression of Otc in this more sensitive qRT-PCR analysis. As expected, siTcf7l2 decreased TCF7L2 mRNA and protein expression markedly, significantly increased HNF4α expression (Figure 9), and also altered the expression of 13 other examined (Figure 9C). For these genes, the log_2_-transformed FCs upon *Tcf7l2* silencing from qRT-PCR had a very high correlation with those from RNA-seq (Pearson *R*^2^: 0.928, *P* < 4 × 10^−8^), thus confirming our RNA-Seq findings (Supplementary Figure S12).

Silencing of HNF4α alone did not affect *Tcf7l2* expression, but significantly altered the expression of multiple genes that were regulated by *Tcf7l2* silencing in the opposite direction, including those involved in lipid synthesis and transport (i.e. *Mttp*, *Aldob* and *Apob*) and glucose metabolism (*Scl2a2*, *Fbp1*) (Figure 9C). These genes contain strong HNF4α binding sites, but are bound only weakly or not at all by TCF7L2. HNF4α also was a significant independent regulator of genes regulating glycine (*Gldc*), arginine (*Asl*) and urea (*Otc*) metabolism, but did not affect the expression of the Wnt gene *Lef1* or *Dgat2*. Preventing the rise of HNF4α following siTcf7l2 using co-silencing experiments confirmed that HNF4α expression is required for the full effect of *Tcf7l2*-silencing on *Aldob, Apob, Asl, Fbp1, Gldc, Mttp* and *Otc*. Silencing *Hnf4α* alone did regulate *Slc2a2*, but in the dual-silencing experiment the expression of this gene was not significantly different when compared to the siTcf7l2 treated cells, suggesting that TCF7L2 is the dominant regulator of *Slc2a2*. Taken together, these data suggest that the balance between HNF4α and TCF7L2 levels is an important determinant of hepatic metabolic gene expression.

## DISCUSSION

The data in current study highlight several important and new concepts related to TCF7L2 activity in hepatoma cells that may be of significance to hepatocyte development and physiology in animals and humans. Using a novel temporal RNA-Seq and ChIP-Seq experimental design, we demonstrate that TCF7L2 regulates over 400 genes in hepatoma cells, and that early gene expression changes (≤18 h post-*Tcf7l2* silencing) and a strong proximal presence of TCF7L2 (peak proximity score > 10), indicate that TCF7L2 directly regulates 149 of these genes in hepatoma cells. TCF7L2 is an integral member of Wnt signaling, and a number of the pathways regulated by *Tcf7l2* silencing in our study have been linked to Wnt activity in numerous tissues and cells, including those related to cell growth, differentiation and cancer (1). However, previous studies have focused on the role of the transcriptional co-activator β-catenin in these pathways, but our study demonstrates that TCF7L2 *per se* may be the key mediator of Wnt signaling in the liver. Surprisingly, our data also provide evidence that TCF7L2 plays a major role in the regulation of metabolism in liver cells, especially lipid, protein/amino-acid and carbohydrate metabolism. While recent studies have explored the role of TCF7L2 in HGP, we show that the metabolic role of TCF7L2 in the liver extends far beyond this single pathway, and that the diverse metabolic role played by TCF7L2 is accomplished via interactions with numerous key transcription factors in the liver, including HNF4α, FOXO1, MYC, CITED2 and LEF1. For the first time, we demonstrate that the balance between TCF7L2 and HNF4α levels determines the expression of numerous metabolic genes in hepatoma cells. Taken together our study significantly broadens our understanding of TCF7L2 biology, and suggests that TCF7L2 is a critical determinant of the hepatic phenotype and, as such, may play an essential role in regulating whole body homeostasis.

A number of studies have examined DNA binding patterns of TCF7L2 in various cell types (9,17–19) in an effort to extrapolate chromatin occupancy to transcriptional activity and, thus, physiological function. While these studies have provided important insights into the potential mechanistic role of TCF7L2 in various cell-types, no study has systematically examined the functional significance of genome-wide TCF7L2 DNA binding on global transcription. To address this, we used a novel temporal RNA- and ChIP-Seq approach to track gene expression changes following transient silencing of *Tcf7l2*. A reduction in *Tcf7l2* mRNA and protein expression, as well as a global reduction in TCF7L2 chromatin occupancy, led to a time-dependent change in the expression of over 400 genes, and we first set out to determine which of these may represent direct TCF7L2 targets. A total of 149 genes that were found to be differentially expressed up to and including 18 h after silencing also had a strong proximal presence of TCF7L2 (defined as a peak proximity score > 10), suggesting direct regulation by TCF7L2.

An important observation from our analysis is that wild-type TCF7L2 appears to primarily act to induce gene expression in hepatoma cells. This was apparent after overlaying genome-wide TCF7L2 binding peaks with siRNA-*Tcf7l2* mediated changes in gene expression. We observed a clear increase in the presence of proximal TCF7L2 ChIP-Seq peaks near genes that were down-regulated by *Tcf7l2* silencing, whereas up-regulated genes were less enriched for proximal TCF7L2 binding. While this is a simplistic view of Wnt/β-catenin mediated transcription, because TCF7L2 cooperates with β-catenin and other transcription factors to regulate target gene expression, recent evidence indicates that TCF7L2 is a predominant mediator of Wnt-mediated transcription and that β-catenin is bound exclusively by TCFs (31). Of note, TCF7L2 binding is not restricted to proximal promoter regions. However, what role relatively distal binding plays in the regulation of gene expression by TCF7L2 is currently unknown. A very recent study suggests that for some transcription factors, proximal and distal binding events may regulate completely different biological processes (32). Our temporal RNA-Seq IPA pathway analyses indicated that early and more directly regulated DEGs tended to be related to cell growth, differentiation and cancer pathways in the hepatoma cells, which is consistent with the role of Wnt signaling in these developmental pathways. As an example of this direct gene regulation by TCF7L2, the classic Wnt target genes *Lef1* and *Axin2* demonstrated early changes in gene expression following *Tcf7l2* silencing, and were bound proximally by TCF7L2. Further, maximal reductions in the expression of *Lef1* and *Axin2* mirrored precisely the maximal reduction in *Tcf7l2* expression and began to recover as *Tcf7l2* expression approached levels observed in scramble-treated cells. We also highlight similar effects at the ornithine aminotransferase (*Oat*) and liver-specific glycogen synthase (*Gys2*) genes, indicating that these metabolic genes also are direct TCF7L2-regulated genes in hepatoma cells.

It is apparent from our study that TCF7L2 has an important and diverse molecular role in hepatoma cells, and that it regulates an array of pathways essential for hepatocyte physiology. Performing IPA analysis on all 406 DEGs identified lipid metabolism and cell growth, differentiation and cancer pathways as most enriched. After analyzing the time-course RNA-Seq data, we identified nine broad pathway categories that were regulated by *Tcf7l2* silencing, and these fit broadly into two major themes—cell growth, differentiation and cancer, and liver metabolism, which included lipid, carbohydrate and amino acid and urea metabolic pathways. As detailed above, the cell growth related pathways appeared earlier in the RNA-Seq time course and may be under more direct control by TCF7L2, whereas metabolic pathways appeared later, suggesting more indirect regulation by TCF7L2, involving several other transcription factors. In general *Tcf7l2* silencing was associated with a gene expression program consistent with reduced cell invasiveness, diminished capacity for liver injury repair and a mesenchymal to epithelial transition (MET) in hepatoma cells. The tumor suppressor gene and epithelial cell marker E-cadherin (*Cdh1*) was markedly up-regulated and the oncogenic transcription factor *Myc* was down-regulated by *Tcf7l2* silencing. The hepatocyte growth factor/c-met (*Met*) and the amphiregulin (*Areg*) genes also were both significantly down-regulated by *Tcf7l2* silencing, and these genes are known to be important regulators of liver injury repair (33,34). These functions are consistent with the general role of Wnt signaling in these pathways (35), but we show for the first time that TCF7L2 is a global regulator of genes regulating these cellular processes in a liver-related cell line.

In addition to these pathways, our data also demonstrate that TCF7L2 is a major regulator of metabolic genes in hepatoma cells. In mature hepatocytes, precise coordination of carbohydrate, lipid, protein/amino-acid and xenobiotic metabolism is paramount, and TCF7L2 mediates many of these pathways, which we show can occur directly or indirectly through cooperation with hepatic transcription factors. A central and novel finding in our study was that *Tcf7l2* has a significant role in hepatic lipid metabolism and transport which, to our knowledge, has not previously been described. In our RNA-Seq experiments, *Tcf7l2* knock-down surprisingly led to significant gene expression changes of many essential lipid-related genes. This included an approximate doubling of *Apob* gene expression after 48 h and an approximate 4-fold increase at 96 h, which was accompanied by a doubling in the expression of the *Mttp* and *Abcg8* genes at 48 h. Given the known roles of these genes in hepatic lipid metabolism (36–38), it is highly likely that reduced *Tcf7l2* expression promotes an increase in cholesterol synthesis, trafficking and secretion from hepatoma cells. While this role of TCF7L2 has not previously been described, one study has demonstrated that the Wnt co-receptor LRP5 regulates plasma cholesterol levels (39). Mice lacking LRP5 expression display increased plasma cholesterol after being fed a high-fat diet, which was caused by reduced hepatic cholesterol clearance, and was likely exacerbated by defects in glucose-stimulated insulin secretion from β-cells (39). In humans, mutations in the gene encoding LRP6 are associated with hyperlipidemia in humans (40). Carriers of the LRP6 mutation had drastically elevated triglycerides and low-density lipoproteins (LDL), were hyperglycemic, and had a higher incidence of T2DM, despite a normal BMI (40). Expression of mutant LRP6 in NIH3T3 cells markedly reduced *Lef1* transcriptional activity, which is consistent with an overall reduction in Wnt signaling, and also the data in our current study, where a significant reduction in *Lef1* expression also was observed. Furthermore, in mice lacking β-catenin, an increase in liver cholesterol was observed (41), but other studies have demonstrated that over-expression of β-catenin increased hepatic triglyceride content (42).

Similar to lipid-metabolism genes, TCF7L2 was also a significant regulator of amino acid metabolism, especially arginine, cysteine, tryptophan, methionine and citrulline metabolism, and also controlled the expression of many enzymes involved in the urea cycle. These pathways are not mutually exclusive, and are connected through common or linked enzymes. For example, arginine is produced from citrulline via the action of argininosuccinate synthetase (ASS1) and argininosuccinate lyase (ASL), the genes of which were both up-regulated following *Tcf7l2* silencing. Further, citrulline is produced from ornithine, which is regulated by the action of ornithine aminotransferase (OAT, encoded by *Oat*), a clear direct TCF7L2 target gene in our study.

In terms of glucose metabolism, a number of studies have demonstrated that native TCF7L2 is a negative regulator of genes controlling gluconeogenesis in hepatocytes in culture and *in vivo* (8–10,16), but we show that the role of TCF7L2 in hepatic glucose metabolism extends beyond gluconeogenesis. Genes regulating glycogen metabolism (*Gys2*), glycolysis (*Aldob* and *Eno2*) and glucose transport (*Slc2a2*) were all affected by *Tcf7l2* silencing in the hepatoma cells, suggesting that TCF7L2 activity is an important determinant of carbohydrate metabolism overall in the liver. Taken together these data describe new metabolic roles for TCF7L2 in the liver that have not previously been described and it will be important to examine whether these metabolic gene expression changes induced by *Tcf7l2* knock-down translate to physiological and metabolic alterations in mature hepatocytes *in vivo.*

A second important and novel finding in our study is that TCF7L2 indirectly regulates gene expression through cooperation with a number of important hepatic transcriptional partners, and this cooperation may explain the diverse metabolic pathways affected by *Tcf7l2* silencing. We found that several master regulators of hepatic gene transcription were differentially regulated by TCF7L2 in the RNA-Seq time-course. These genes included those encoding HNF4α, MYC, LEF1, CITED2, MIXL1 and FOXO1. Because of its clear role in regulating hepatic metabolic gene transcription, we focused our analysis on the relationship between TCF7L2 and HNF4α. We previously documented an increase in *Hnf4a* mRNA following *Tcf7l2* silencing (9) and a molecular and physical relationship between these two important transcription factors has been documented by others (8,19,43). A recent report showed that TCF7L2 isoforms co-precipitate with HNF4α and modify its nuclear expression (8). In our study, silencing *Tcf7l2* not only increased *Hnf4a* mRNA and protein levels, but also led to an increase in HNF4α chromatin occupancy genome-wide. HNF4α binding was focused around genes that were up-regulated by *Tcf7l2* silencing, indicating a potential cooperative relationship between these two factors.

Many metabolic genes that were differentially expressed in our dataset and were bound by HNF4α included those important for gluconeogenesis (i.e. *Fbp1*, *G6pc*) and cholesterol and triglyceride metabolism (i.e. *Dgat2* and *Apob*), which is consistent with a role for HNF4α in regulating these pathways in the liver. To examine this more closely, we performed co-silencing experiments of TCF7L2 and HNF4α, and examined the expression of several metabolic genes in the hepatoma cells. The data from these experiments highlight that HNF4α is indeed required for the effect of TCF7L2 silencing on the *Mttp* and *Apob* genes, and the independent regulation of liver fat genes by HNF4α also has been demonstrated in HNF4α liver-specific null mice (44). Knock-out of HNF4α in mouse liver leads to massive liver triglyceride accumulation, which is the result of an almost complete absence of *Mttp* and *Apob* expression and leads to reduced VLDL-mediated secretion of triglycerides (44). Similarly, we also show that HNF4α is required for the effect of TCF7L2 on amino acid and urea cycle genes, particularly *Asl* and *Otc*, and that it can regulate the expression of these genes independently of TCF7L2. The independent role of HNF4α in this pathway is consistent with a previous report demonstrating that liver-specific disruption of HNF4α drastically reduces *Otc* expression and disrupts ureagenesis (45). For glucose metabolism, silencing HNF4α also blunted the effect of TCF7L2 silencing on the expression of *Aldob and Fbp1*, but did not appear to be required for the effect of TCF7L2 on *G6pc* and *Slc2a2* expression. These data indicate that (i) the balance between HNF4α and TCF7L2 expression plays a defining role in the regulation of a subset of metabolic genes in liver cells that will require further exploration genome-wide, and (ii) TCF7L2 likely interacts with several additional transcription factors to regulate gene expression in these cells. Of particular interest in this regard is FOXO1, as this factor has been shown to interact with TCF7L2 to regulate metabolic gene expression in mouse liver *in vivo* (10).

The changes in the expression of many of the cooperating transcription factors in our study persisted at the 96 h end-point of our RNA-Seq study, which may explain the persistent changes in some metabolic genes; the expression of *Hnf4α*, *Foxo1*, *Cited2* and *Mixl1* remained differentially expressed when *Tcf7l2* had returned to near basal levels. These findings may have arisen directly from the residual depletion of TCF7L2 mRNA and protein at 96 h, or indirectly as a result of the cascading nature of gene expression changes, such that a factor that was altered early in our time-course affected the downstream expression of a gene later on that persisted beyond the period of maximal *Tcf7l2* silencing. The expression of the important developmental transcription factor *Mixl1* exponentially decreased during the entire 96 h time-course and did not recover which also may indicate that some gene expression changes induced by siTcf7l2 may be relatively permanent and/or extremely slow to recover, which may further contribute to persistent secondary gene expression changes.

In summary, the results of our study provide novel and important insights into the mechanisms of gene regulation by TCF7L2 in hepatoma cells and further advance our understanding of how TCF7L2 may impact the cellular and metabolic pathways that are important to liver cell development, cancer and hepatic metabolic disorders. We highlight that TCF7L2 has a diverse role in hepatoma cells and, through direct and indirect gene regulation, regulates multiple pathways. Future studies conducted *in vivo* will be needed to validate these findings, and similar experiments in other tissues and cells that are important for metabolic diseases will be required.

## ACCESSION NUMBERS

Raw and processed RNA-seq and ChIP-seq data, including the H4IIE-specific transcriptome file in gtf-format, have been deposited to the NCBI under accession number GSE53862.

## SUPPLEMENTARY DATA

Supplementary Data are available at NAR Online.

SUPPLEMENTARY DATA
