# Supplementary material for: The mechanisms of genome-wide target gene regulation by TCF7L2 in liver cells
Source: Nucleic Acids Res. 2014 Nov 20;42(22):13646–61. doi: 10.1093/nar/gku1225 (PMC4267646; doi:10.1093/nar/gku1225)
Supplement: SUPPLEMENTARY DATA [file supp_42_22_13646__index.html]

The mechanisms of genome-wide target gene regulation by TCF7L2 in liver cells — The mechanisms of genome-wide target gene regulation by TCF7L2 in liver cells — SUPPLEMENTARY DATA 

# The mechanisms of genome-wide target gene regulation by TCF7L2 in liver cells

## SUPPLEMENTARY DATA

**Files in this Data Supplement:**

- SUPPLEMENTARY DATA
- SUPPLEMENTARY DATA
