# Supplementary material for: The mechanisms of genome-wide target gene regulation by TCF7L2 in liver cells
Source: Nucleic Acids Res. 2014 Nov 20;42(22):13646–61. doi: 10.1093/nar/gku1225 (PMC4267646; doi:10.1093/nar/gku1225)
Supplement: SUPPLEMENTARY DATA [file supp_gku1225_nar-00977-v-2014-File011.pdf]

SUPPLEMENTARY METHODS

Quantitative RT-PCR assays

The following TaqMan® Gene Expression Assays (Applied Biosystems) were used in this study (gene – catalog number):
Controls: *Actb* - Rn00667869_m1, *B2m* – Rn00560865_m1, and *Hmbs* - Rn00565886_m1.
Targets: *Aldob* – Rn01768292_m1, *Apob* - Rn01499054_m1, *Asl* - Rn01480437_g1, *Cps1* - Rn00567109_m1, *Dgat2* – Rn01506787_m1, *Fbp1* – Rn00561189_m1, *G6pc* – Rn00689876_m1, *Gldc* - Rn01501761_m1, *Hnf4a* - Rn00573309_m1, *Lef1* - Rn01522501_m1, *Mttp* - Rn01522963_m1, *Oat* – Rn00755544_m1, *Otc* - Rn00565169_m1, *Slc2a2* - Rn00563565_m1, and *Tcf7l2* - Rn01411019_m1.

Experimental time line

The schematic time line for the sampling of H4IIE hepatoma cells for ChIP-seq and RNA-seq analysis over the *Tcf7l2* silencing time course is depicted in **Figure 1.**

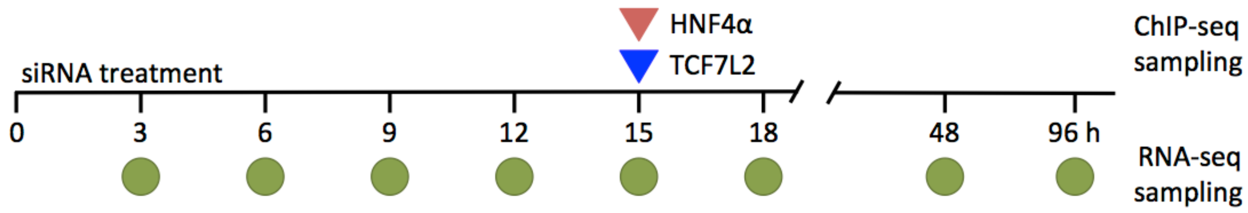

Figure 1. Schematic depiction of the time course for *Tcf7l2* silencing and sampling for ChIP-seq and RNA-seq analysis.

RNA-Seq sample preparation

At the indicated time-points (see Figure 1 above), media was removed and cells were washed two times with ice-cold PBS before 500 µl of Trizol was added directly to the cells. The Trizol solution was pipetted several times until all cells were removed from the surface of the plate, and then the cell solution was placed into a -80°C freezer until all samples had been collected for the duration of the time-course. RNA-Seq library preparation was carried out using the Illumina TruSeq RNA-Seq kit as described by the manufacturer (Low-throughput method), except that homemade SPRI beads were used throughout for DNA clean-ups. Input RNA for library preparation was normalized within each time-point and final libraries were validated using HS DNA Bioanalyzer chips. Samples were quantified using pico-green and six indexed samples were pooled per lane of the Illumina HiSeq 2000 (50 bp, single-end) and run at the Genomics Core Facility at UTSW, Dallas.

ChIP-Seq sample preparation

ChIP-Seq was performed on scramble and siTcf7l2 treated cells in duplicate (for TCF7L2) and each sequenced sample represented a pool of 2 independent immunoprecipitations, each consisting of between 10 and 20 µg of chromatin. Chromatin was prepared from approximately 2.5 x 10^7 siTcf7l2 or scramble electroporated cells and sheared with MNase using the SimpleChIP Enzymatic Chromatin IP Kit (Cell Signaling Technology). Chromatin was fragmented such that the majority of the chromatin was between 150 bp and 900 bp and each aliquot was incubated with either 5 µl of the anti-TCF7L2 antibody or 1 µl of the anti-HNF4α antibody. A sample consisting of 2% of the total input chromatin was removed from each and served as the sequencing control (see data analysis for more details). Following an overnight incubation with antibody, the immunoprecipitated material was purified as described by the manufacturer (Cell Signaling Technology), except that replicate immunoprecipitations were pooled at the final Qiagen DNA purification step. ChIP-Seq libraries were generated using a combination of Illumina TruSeq adapters/indexes and a Kapa Library Preparation Kit (Kapa Biosystems, Woburn, MA, USA; cat # KK8200). The protocol deviated from the standard Illumina TruSeq ChIP-Seq and Kapa library preparation protocols in some important ways. End-repair was performed on approximately 5 – 10 ng of ChIP sample and adenylation and adapter ligation were performed as described in the Kapa protocol, except Qiagen Minelute purifications (Qiagen, Valencia, CA, USA) were utilized in between each step. Adapter ligation was carried out using stock Illumina adapters and Kapa T4 ligase at 30°C for 10 minutes, and this was followed by a single DNA clean-up using homemade SPRI beads. A minimal PCR amplification of the adapted material was performed prior to size selection to convert y-shaped adapters to double-stranded DNA for more reliable electrophoresis and size selection. This PCR consisted of 5 cycles with the Illumina PCR primer cocktail and Kapa HiFi HotStart Polymerase ReadyMix (cat # KK2601). Following a Qiagen Minelute purification, the adapted material was size selected on a 2% agarose gel before a final PCR amplification of between 8 – 12 cycles and SPRI bead clean-up. Final libraries were checked for size using Bioanalyzer HS DNA or DNA 1000 chips and enrichment was confirmed in the final product using *Axin2*, *Lef1* (for TCF7L2) and *Apoc3* (for HNF4α) loci using real-time PCR.

Data analysis

Defining the H4IIE-specific transcriptome

To account for the relatively incomplete nature of the rn4 reference transcriptome (compared to human and mouse genomes), we first defined the H4IIE-specific transcriptome from a large pool of 213.4 million RNA-seq reads originating from a separate experiment (24 h treatment of TCF7L2 or scramble siRNA, 3

biological replicates each). Because the rn4 genome has a short gap within the *Tcf7l2* gene that includes part of an exon - rendering RNA-seq read alignment at this loci ineffectual - it was partially filled using rat EST sequences, resulting in updating the reference exon start location from chr1:262,210,923 to 262,210,894. A corresponding fix was also introduced into the UCSC reference transcriptome for that *Tcf7l2* exon. The pooled reads were mapped, using Tophat version 2.0.4 (with underlying Bowtie version 0.12.8) (1,2), against UCSC known transcripts for rn4, supplemented with additional transcripts from Ensembl, and allowing for the identification of novel exon-exon junctions for the reference genome (rn4). Essential Tophat command arguments were: `--library-type fr-unstranded --prefilter-multihits --no-coverage-search -G <UCSC+Ensembl known transcripts>`. Transcript mappings were further processed using Cufflinks package version 2.0.2 (3) with essential command arguments for Cufflinks: `--frag-bias-correct --multi-read-correct --upper-quartile-norm -g <UCSC+Ensembl known transcripts> --library-type fr-unstranded`, and for Cuffcompare: `-r <UCSC+Ensembl known transcripts> -R -d 20 -V <.gff output of Cufflinks>`. The resulting initial transcriptome was cleaned by removing all transcripts that were identified as strandless (in Cuffcompare class codes u, o, x, and, if also single-exon, i), likely artifacts (Cuffcompare class codes e, p, r, s and c), or fully silent (FPKM=0) in this large pooled read set with roughly 6 times more reads than in the subsequent time course RNA-seq samples. The transcripts that did not represent any known rn4 gene were then analyzed against mouse (mm10), human (hg19), as well as an updated rat reference transcript sequences using discontiguous megablast with stringent cutoffs to provide official gene symbols additional gene identification. The resulting H4IIE-specific transcriptome that contains 22125 transcripts representing 15768 genes, out of which 2112 (9.5%) transcripts representing 861 (5.5%) genes remained without an official gene symbol and is made available as part of the GEO accession GSE53862.

Defining the consensus peak locations

The main motivation behind the consensus summit calculation method (4) is to answer to the difficult question of which ChIP-seq peaks represent “the same” binding sites in different samples for the same transcription factor. To better distinguish nearby binding sites from each other, the method resorts to, instead of the peaks themselves that typically are a few hundred bp wide, the punctate peak summits, i.e. the single nucleotide positions within each peak where the ChIP-seq signal is at maximum. The main processing steps include 1) collecting all peak summit locations from all ChIP-seq samples to be compared, 2) collapsing that whole summit location data set into a single vector of summit locations per chromosome, 3) inspecting the local density of the summit locations to determine, with suitable cutoffs and other parameters (essentially, triangular density with bandwidth of 20 bp and therein, local maxima

detection with span of 100 bp), which individual summits likely represent the same binding site, thereby 4) forming the set of consensus summit locations that accurately capture all the binding events that were identified by the original ChIP-seq peak calling in any of the included samples. Subsequent processing assigns each consensus summit location in each sample the desired numerical values, e.g. FE (with the help of bedgraph pileup files saved during the original peak calling and extracting the scores using the MACS2 bdgcmp program (https://github.com/taoliu/MACS/)), regardless of whether a peak was originally identified at that location or not, thereby enabling easy and reliable comparisons across the entire dataset. Finally, for uses were wider, peak-like regions are required or beneficial, the consensus summit locations were extended by ±100 bp but without allowing overlaps between adjacent summits less than 200 bp apart. At best this method is when there are more than 2 samples to compare; our motivation to use it also here was to be able to compare peak FE values across the entire peak set, including those locations were only one sample had an original peak.

Integration of DEGs, ChIP-seq peaks and binding motifs

'Peak proximity scores' were calculated, using the algorithm introduced by Ouyang et al. (5), on our TCF7L2 and HNF4α ChIP-seq data in conjunction with the RNA-seq–identified DEGs over the siTcf7l2-treatment time-course. Briefly, for each peak-gene pair (the gene represented by its TSS), a geometrically decaying distance was multiplied by the peak FE, and these individual scores summed for that gene (TSS). In the calculations for the geometrically decaying distance, we defined the critical parameter 'd0' as 50,000 bp in order to restrict, in practice, any contribution from a peak to the gene-wise score to up to about ± 200 kb. Given the tolerable increase in computation time, we nonetheless performed the calculations on all peaks that were up to 1 Mb from the gene TSS.

The known motif analysis using HOMER version 4.3 was performed using a set of known motifs supplied with HOMER that was first reduced to remove similar motifs using a similarity cutoff 0.80 (6).

References

1. Langmead, B., Trapnell, C., Pop, M. and Salzberg, S.L. (2009) Ultrafast and memory-efficient alignment of short DNA sequences to the human genome. *Genome Biol*, **10**, R25.
2. Kim, D., Pertea, G., Trapnell, C., Pimentel, H., Kelley, R. and Salzberg, S.L. (2013) TopHat2: accurate alignment of transcriptomes in the presence of insertions, deletions and gene fusions. *Genome Biol*, **14**, R36.
3. Trapnell, C., Hendrickson, D.G., Sauvageau, M., Goff, L., Rinn, J.L. and Pachter, L. (2013) Differential analysis of gene regulation at transcript resolution with RNA-seq. *Nat Biotechnol*, **31**, 46-53.

4. Tuoresmäki, P., Väisänen, S., Neme, A., Heikkinen, S. and Carlberg, C. (2014) Patterns of genome-wide VDR locations. *PLoS One*, **9**, e96105.
5. Ouyang, Z., Zhou, Q. and Wong, W.H. (2009) ChIP-Seq of transcription factors predicts absolute and differential gene expression in embryonic stem cells. *Proc Natl Acad Sci U S A*, **106**, 21521-21526.
6. Heinz, S., Benner, C., Spann, N., Bertolino, E., Lin, Y.C., Laslo, P., Cheng, J.X., Murre, C., Singh, H. and Glass, C.K. (2010) Simple combinations of lineage-determining transcription factors prime cis-regulatory elements required for macrophage and B cell identities. *Mol Cell*, **38**, 576-589.

SUPPLEMENTARY FIGURE LEGENDS

Supplementary Figure S1. Validation of the HNF4α ChIP-seq antibody. The mouse monoclonal antibody (clone H1415, cat # PP-H1415-00, R&D Systems, Minneapolis, MN, USA) used in HNF4α ChIP-Seq analysis was validated using ChIP-PCR with an amplicon adjacent to an established binding site at the rat apolipoprotein-C-III (*Apoc3*) gene promoter as schematically depicted in **A**). The ChIP-PCR results in **B**) show the highly increased signal from the HNF4α site in *Apoc3* promoter but minimal signals from the same site with MYC-tag antibody (clone 71D10, cat #2278, Cell Signaling Technology) or, with the HNF4α antibody, from a site in *Slc2a4* (also called *Glut4*) promoter that is known not to bind HNF4α.

Supplementary Figure S2. High concordance between TCF7L2 ChIP-seq replicates, and the reduction of TCF7L2 binding upon *Tcf7l2* silencing. A) The between-replicate overlaps of the high-confidence peak sets (FDR < 0.01) identified in the TCF7L2 ChIP-seq samples using MACS version 2 (Venn diagrams left of the arrow) for Scr (left) and siTcf7l2 (right), and the between-treatment overlap upon the subsequent peak calling on merged replicates (Venn diagram right of the arrow). **B**) Comparison of TCF7L2 ChIP-seq peak fold enrichments between the Scr and siTcf7l2 samples at all TCF7L2 binding locations. The intensity of the blue color is indicative of how densely the points, representing peaks, appear at a given region of the plot. **C**) TCF7L2 occupancy at the *Tcf7l2* gene locus. Normalized TCF7L2 ChIP-seq read accumulation tracks are displayed for Input, Scr and siTcf7l2 samples collected at 15 h siRNA treatment. High-confidence (FDR < 0.01) ChIP-seq peaks are shown under the Scr and siTcf7l2 tracks with bars whose coloring (from pale yellow to red) indicates the peak size (fold enrichment over Input). Merged peaks are displayed as black bars above the gene body track in blue. **D**) TCF7L2 ChIP-

PCR of an established binding site at the rat *Axin2* gene promoter and a negative control region in *Slc2a4* (also called *Glut4*) gene promoter.

Supplementary Figure S3. TCF7L2 binds multiple metabolically relevant genes in hepatocytes. On the left, ChIP-seq-derived occupancy of TCF7L2, and on the right, the change in gene expression due to siTcf7l2 silencing for **A**) *Pdk1*, **B**) *Aldoa*, **C**) *Dgat2*, and **D**) *Fbp1* and *Fbp2*. On the left panels, normalized TCF7L2 ChIP-seq read accumulation tracks are displayed for Input, Scr and siTcf7l2 samples collected at 15 h siRNA treatment. High-confidence (FDR < 0.01) ChIP-seq peaks are shown under the Scr and siTcf7l2 tracks with bars whose coloring (from pale yellow to red) indicates the peak size (fold enrichment over Input). Merged peaks are displayed as black bars above the gene body track in blue. On the right panels, the RNA-seq-derived gene expression is expressed over the time course as log2 of fold change (siTcf7l2/Scr, with 95% confidence intervals for both siTcf7l2 (blue for down-regulation and red for up-regulation) and Scr (gray)). Statistical significance (Cuffdiff q-value) at a given time point is indicated by stars (*q < 0.05; **q < 0.01; ***q < 0.001).

Supplementary Figure S4. TCF7L2 binds some, but not all transcription factor genes with altered expression upon *Tcf7l2* silencing. ChIP-seq-derived occupancy of TCF7L2 at the loci of **A**) *Mixl1*, **B**) *Foxo1*, **C**) *Myc*, and **D**) *Cited2*. Normalized TCF7L2 ChIP-seq read accumulation tracks are displayed for Input, Scr and siTcf7l2 samples collected at 15 h siRNA treatment. High-confidence (FDR < 0.01) ChIP-seq peaks are shown under the Scr and siTcf7l2 tracks with bars whose coloring (from pale yellow to red) indicates the peak size (fold enrichment over Input). Merged peaks are displayed as black bars above the gene body track in blue.

Supplementary Figure S5. Increase in HNF4α expression and chromatin occupancy upon *Tcf7l2* silencing. H4IIE cells were treated with scrambled (Scr) or *Tcf7l2*-specific (siTcf7l2) siRNA and sampled at 24 and 48 h for protein and at 15 h for chromatin occupancy analysis. **A**) Increase of HNF4α after siTcf7l2 treatment demonstrated by Western blotting. GAPDH was used as loading control, and a representative blot with two replicates is shown. **B**) Quantitation of HNF4α protein from the two separate silencing and Western blot experiments, each with two replicate electroporations (A), relative to GAPDH and expressed as means ± SEM. *p < 0.05; **p < 0.01.

Supplementary Figure S6. Decrease in HNF4α expression upon TCF7L2 overexpression. Human wild-type TCF7L2 and eGFP control were over-expressed in human Hep3B cells using adenovirus infected at a multiplicity of infection (MOI) of 50, and harvested 72 h later for RNA and protein analysis. **A**) Decrease in *HNF4A* gene expression upon TCF7L2 overexpression demonstrated by quantitative RT-PCR measurement. Results were normalized to *B2M* expression and are expressed as means ± SEM

relative to the mean of Ad-eGFP (n = 3 independent infection experiments, each with two replicate wells). *p < 0.05. **B**) Decrease in HNF4α protein after TCF7L2 overexpression demonstrated by Western blotting. GAPDH was used as loading control. **C**) Quantitation of HNF4α protein from the Western blots, relative to GAPDH and expressed as means ± SEM (n = 2 independent experiments, each with two replicate infections).

Supplementary Figure S7. TCF7L2 and HNF4α chromatin occupancy at the *Hnf4a* locus upon *Tcf7l2* silencing and *de novo*-identified consensus binding sequence. **A**) Normalized TCF7L2 and HNF4α ChIP-seq read accumulation tracks are shown for Input, Scr and siTcf7l2 samples as indicated. High-confidence (FDR < 0.01) ChIP-seq peaks are shown under the Scr and siTcf7l2 tracks with bars whose coloring (from pale yellow to red) indicates the peak size (fold enrichment over Input). Merged peaks are displayed as black bars under the respective siTcf7l2 peak tracks. **B**) The best *de novo* binding motif identified within ±100 bp of HNF4α ChIP-seq peak summits using HOMER.

Supplementary Figure S8. **HNF4α preferentially binds near genes up-regulated by the siTcf7l2 treatment.** HNF4α peak proximity scores for each of the full set of 406 differentially expressed genes (DEG) were calculated and plotted together with DEG log2 of fold change (FC, siTcf7l2/Scr) values. **A**) For each time point, the genes were ordered according to their FC and plotted against the peak proximity score running means over each consecutive set of 100 genes. Time points are color coded as indicated. **B**) For each gene, the gene expression changes over the time course were summarized as cumulative FC values summed over those time points at which the gene was statistically significantly affected, considering either all time points (‘All DEGs’) or the indicated subset, and plotted against the respective HNF4α peak proximity score. ‘Late’ genes include all genes that are DEGs at either of the late time points, irrespective of their status during the ‘Early’ time points. ‘Late-only’ are those that are DEGs exclusively at either of the late time points. Blue dots indicate negative and red positive sum of significant FCs. In both A) and B), the horizontal dashed line at peak proximity score 5 indicates the chosen cut-off for defining a gene likely to be a direct target of HNF4α.

Supplementary Figure S9. **TCF7L2 and HNF4α ChIP-seq read densities at merged binding locations upon Scr and siTcf7l2 treatment.** The ChIP-seq read densities ±1 kb around the 11793 merged TCF7L2 and HNF4α peak summits were plotted using ngsplot tool. Clustering is based on the difference between the Scr sample for TCF7L2 and the siTcf7l2 sample for HNF4α. Background read densities (Input sample) are shown on the left. The intensity of the red color indicates the density of ChIP-seq reads.

Supplementary Figure S10. HNF4α occupation at the loci of metabolic and transcription factor genes. ChIP-seq-derived occupancy of HNF4α at the loci of metabolically relevant genes **A**) *Pdk1*, **B**) *Aldoa*, **C**) *Dgat2* and **D**) *Fbp1* and *Fbp2*, and transcription factor genes **E**) *Mixl1*, **F**) *Foxo1*, **G**) *Myc* and **H**) *Cited2*. Normalized HNF4α ChIP-seq read accumulation tracks are displayed for Scr and siTcf7l2 samples collected at 15 h siRNA treatment. High-confidence (FDR < 0.01) ChIP-seq peaks are shown under the Scr and siTcf7l2 tracks with bars whose coloring (from pale yellow to red) indicates the peak size (fold enrichment over Input). Merged peaks are displayed as black bars above the gene body track in blue.

Supplementary Figure S11. HNF4α occupation at the loci of *Tcf7l2*, Wnt/β-catenin and metabolic genes. ChIP-seq-derived occupancy of HNF4α at the loci of **A**) *Tcf7l2*, classic Wnt/β-catenin-pathway genes **B**) *Axin2*, **C**) *Gys2*, **D**) *Lef1* and **E**) *Oat*. Normalized HNF4α ChIP-seq read accumulation tracks are displayed for Scr and siTcf7l2 samples collected at 15 h siRNA treatment. High-confidence (FDR < 0.01) ChIP-seq peaks are shown under the Scr and siTcf7l2 tracks with bars whose coloring (from pale yellow to red) indicates the peak size (fold enrichment over Input). Merged peaks are displayed as black bars above the gene body track in blue.

Supplementary Figure S12. **Correlation between the RNA-seq and qRT-PCR results.** The effects of 48 hour siRNA-mediated *Tcf7l2* silencing for the expression of each indicated gene, derived from RNA-seq (*x-axis*) and qRT-PCR analysis (*y-axis*) on independent samples, are expressed as log2 of fold changes (siTcf7l2/Scr; blue for down-regulation and red for up-regulation). Pearson correlation test R^2 and p-value are indicated as an inset at the upper left corner of the plot.

Figure S1

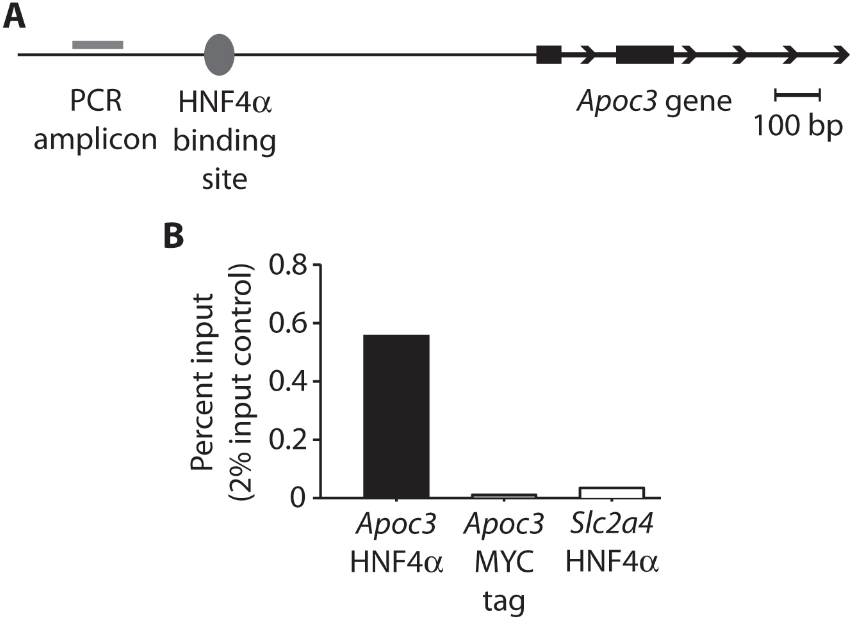
A
PCR amplicon
HNF4α binding site
Apoc3 gene
100 bp
B
0.8
0.6
0.4
0.2
0
Percent input (2% input control)
Apoc3 HNF4α
Apoc3 MYC tag
Slc2a4 HNF4α

Figure S2

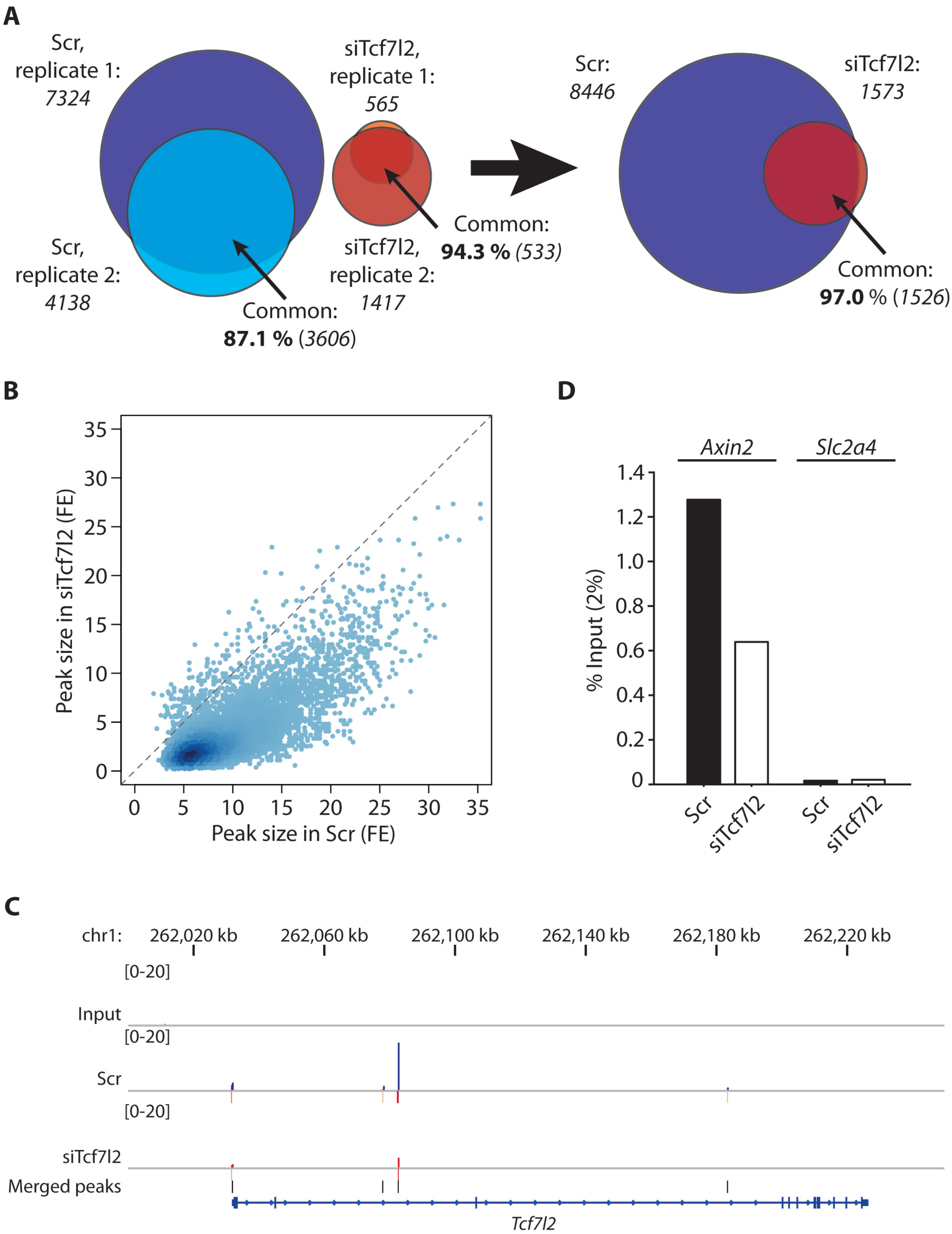

A
Scr, replicate 1: 7324
Scr, replicate 2: 4138
Common: 87.1 % (3606)
siTcf7l2, replicate 1: 565
siTcf7l2, replicate 2: 1417
Common: 94.3 % (533)
Scr: 8446
siTcf7l2: 1573
Common: 97.0 % (1526)
B
Peak size in siTcf7l2 (FE)
Peak size in Scr (FE)
0
5
10
15
20
25
30
35
D
Axin2
Slc2a4
% Input (2%)
1.4
1.2
1.0
0.8
0.6
0.4
0.2
0
Scr
siTcf7l2
Scr
siTcf7l2
C
chr1:
262,020 kb
262,060 kb
262,100 kb
262,140 kb
262,180 kb
262,220 kb
[0-20]
Input
[0-20]
Scr
[0-20]
siTcf7l2
Merged peaks
Tcf7l2

Figure S3

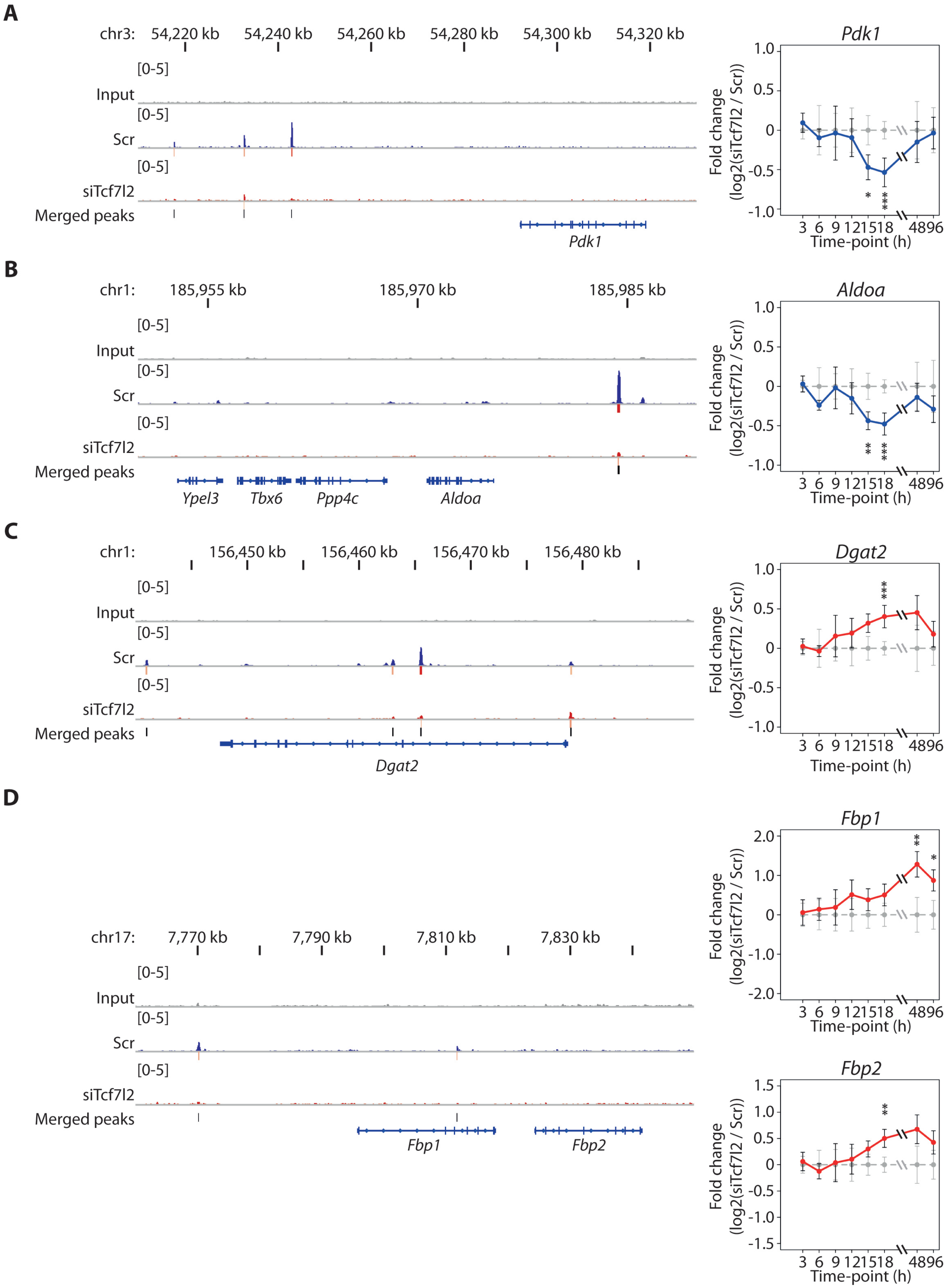
A
chr3:
54,220 kb
54,240 kb
54,260 kb
54,280 kb
54,300 kb
54,320 kb
[0-5]
Input
Scr
siTcf7l2
Merged peaks
Pdk1
B
chr1:
185,955 kb
185,970 kb
185,985 kb
Ypel3
Tbx6
Ppp4c
Aldoa
C
156,450 kb
156,460 kb
156,470 kb
156,480 kb
Dgat2
D
chr17:
7,770 kb
7,790 kb
7,810 kb
7,830 kb
Fbp1
Fbp2
Fold change
(log2(siTcf7l2 / Scr))
Time-point (h)
3 6 9 12 15 18 48 96

Figure S4

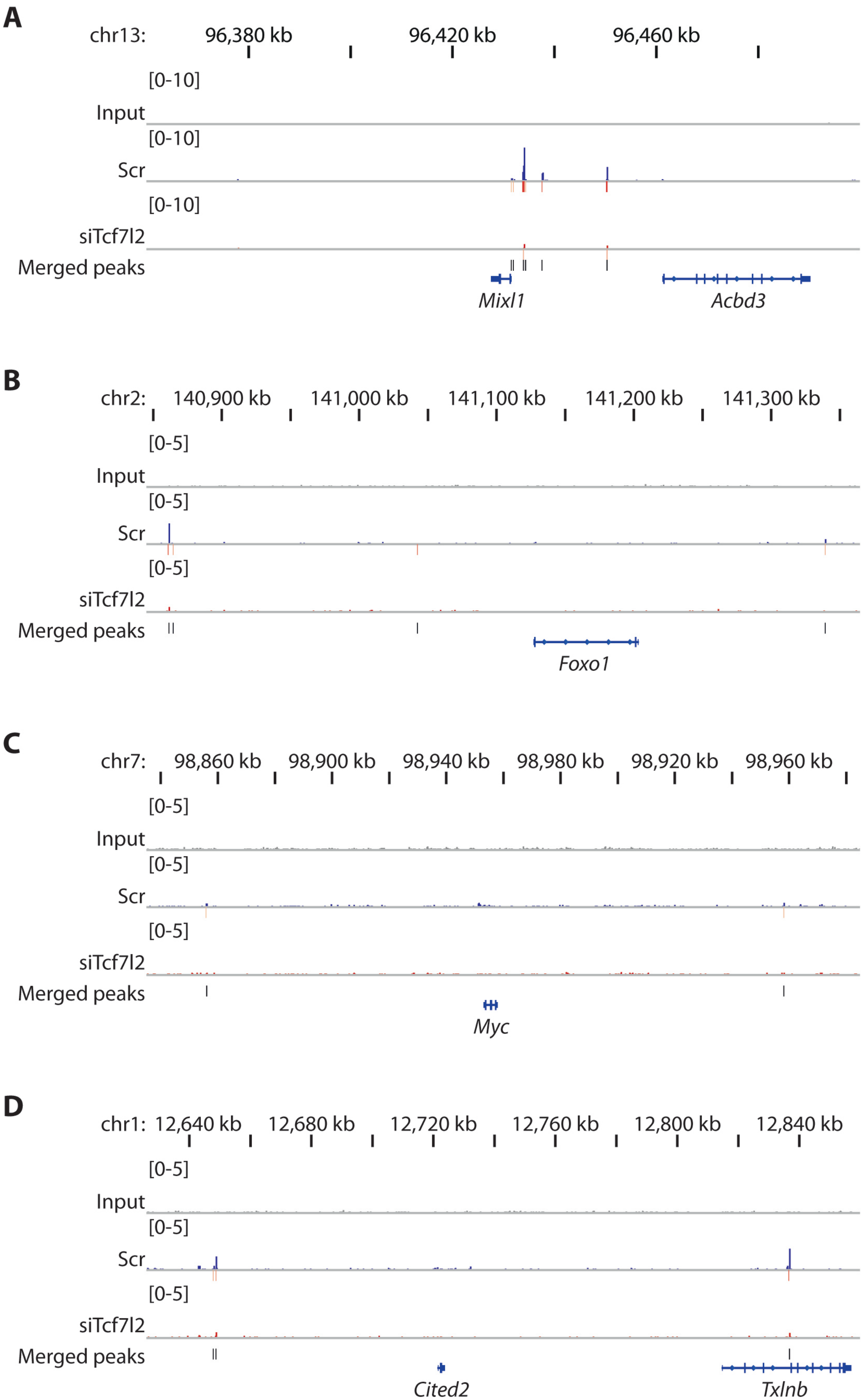
A
chr13:
96,380 kb
96,420 kb
96,460 kb
[0-10]
Input
[0-10]
Scr
[0-10]
siTcf7l2
Merged peaks
Mixl1
Acbd3
B
chr2:
140,900 kb
141,000 kb
141,100 kb
141,200 kb
141,300 kb
[0-5]
Input
[0-5]
Scr
[0-5]
siTcf7l2
Merged peaks
Foxo1
C
chr7:
98,860 kb
98,900 kb
98,940 kb
98,980 kb
98,920 kb
98,960 kb
[0-5]
Input
[0-5]
Scr
[0-5]
siTcf7l2
Merged peaks
Myc
D
chr1:
12,640 kb
12,680 kb
12,720 kb
12,760 kb
12,800 kb
12,840 kb
[0-5]
Input
[0-5]
Scr
[0-5]
siTcf7l2
Merged peaks
Cited2
Txlnb

Figure S5

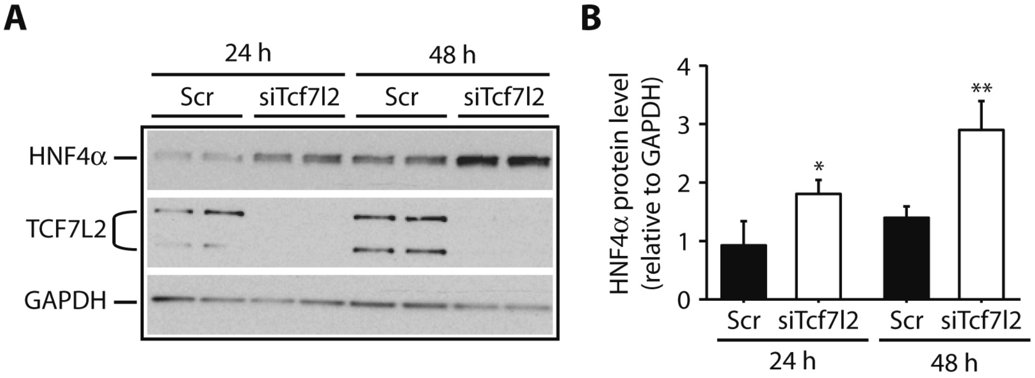
A
24 h
48 h
Scr
siTcf7l2
Scr
siTcf7l2
HNF4α
TCF7L2
GAPDH
B
HNF4α protein level
(relative to GAPDH)
4
3
2
1
0
*
**
Scr
siTcf7l2
Scr
siTcf7l2
24 h
48 h

Figure S6

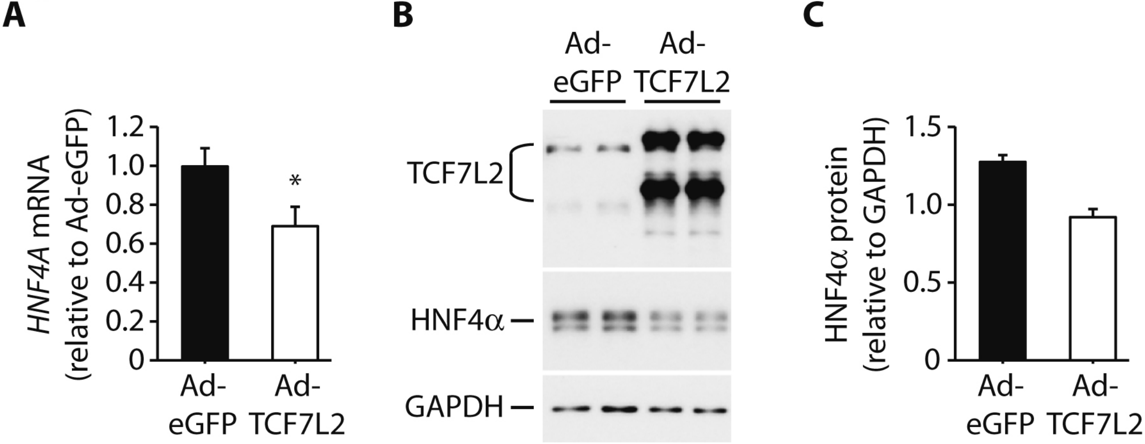
A
HNF4A mRNA (relative to Ad-eGFP)
1.2
1.0
0.8
0.6
0.4
0.2
0
*
Ad-eGFP
Ad-TCF7L2
B
Ad-eGFP
Ad-TCF7L2
TCF7L2
HNF4α
GAPDH
C
HNF4α protein (relative to GAPDH)
1.5
1.0
0.5
0
Ad-eGFP
Ad-TCF7L2

Figure S7

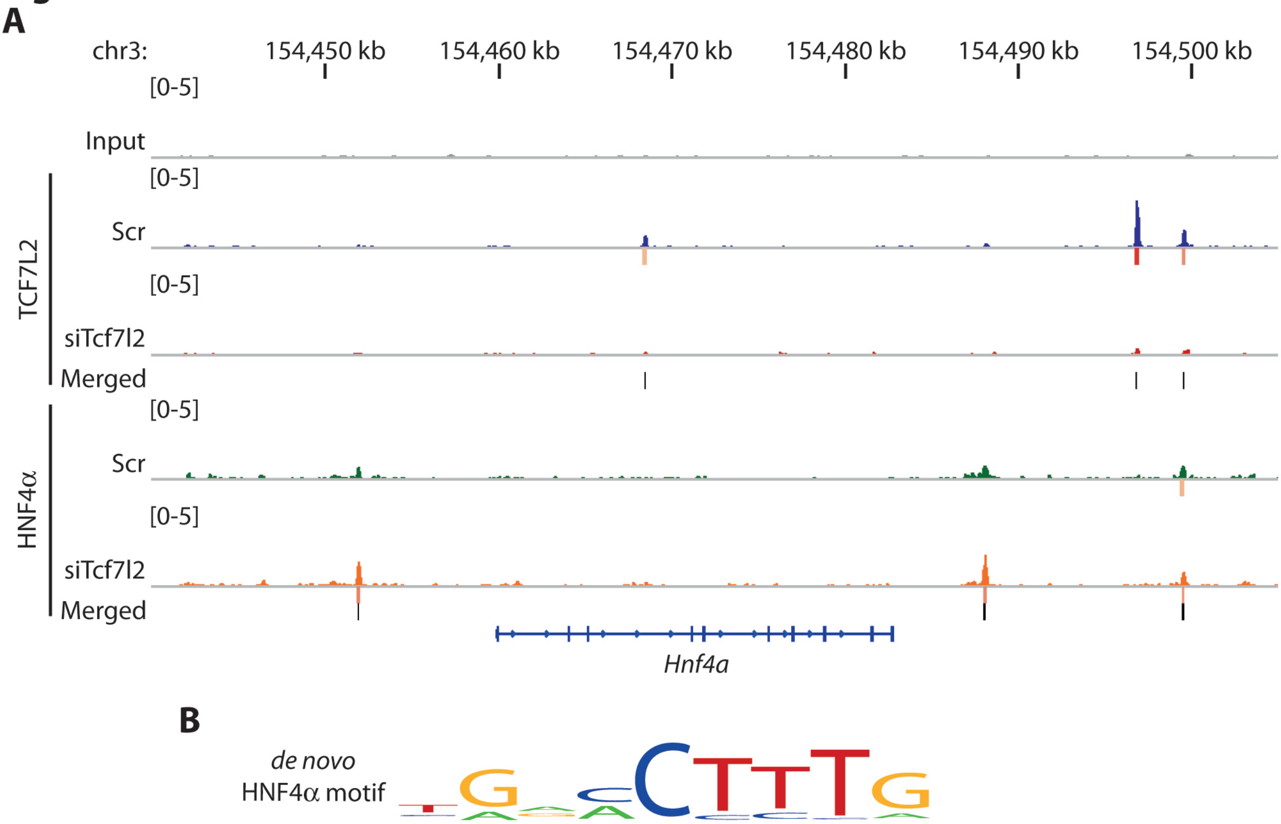
A
chr3:
154,450 kb
154,460 kb
154,470 kb
154,480 kb
154,490 kb
154,500 kb
[0-5]
Input
[0-5]
Scr
[0-5]
siTcf7l2
Merged
TCF7L2
[0-5]
Scr
[0-5]
siTcf7l2
Merged
HNF4α
Hnf4a
B
de novo
HNF4α motif

Figure S8

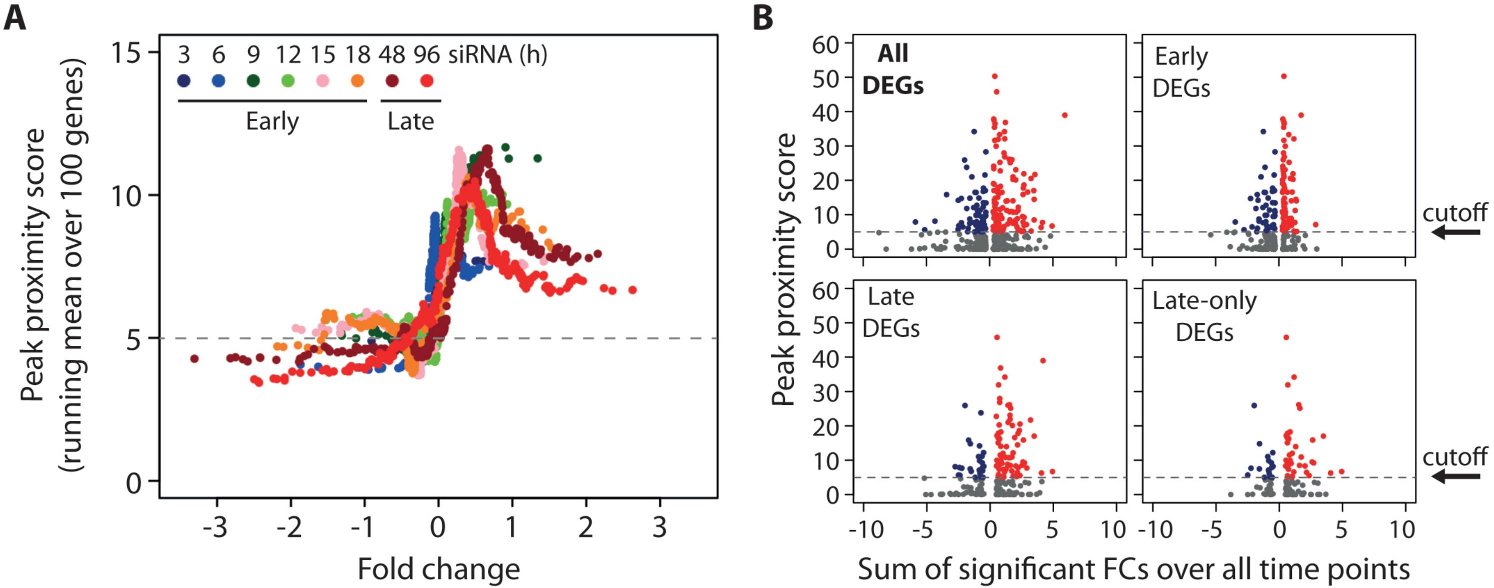
A
B
3 6 9 12 15 18 48 96 siRNA (h)
Early
Late
Peak proximity score (running mean over 100 genes)
Fold change
15
10
5
0
-3
-2
-1
0
1
2
3
All DEGs
Early DEGs
Late DEGs
Late-only DEGs
Peak proximity score
Sum of significant FCs over all time points
cutoff
cutoff
60
50
40
30
20
10
0
-10
-5
0
5
10

Figure S9

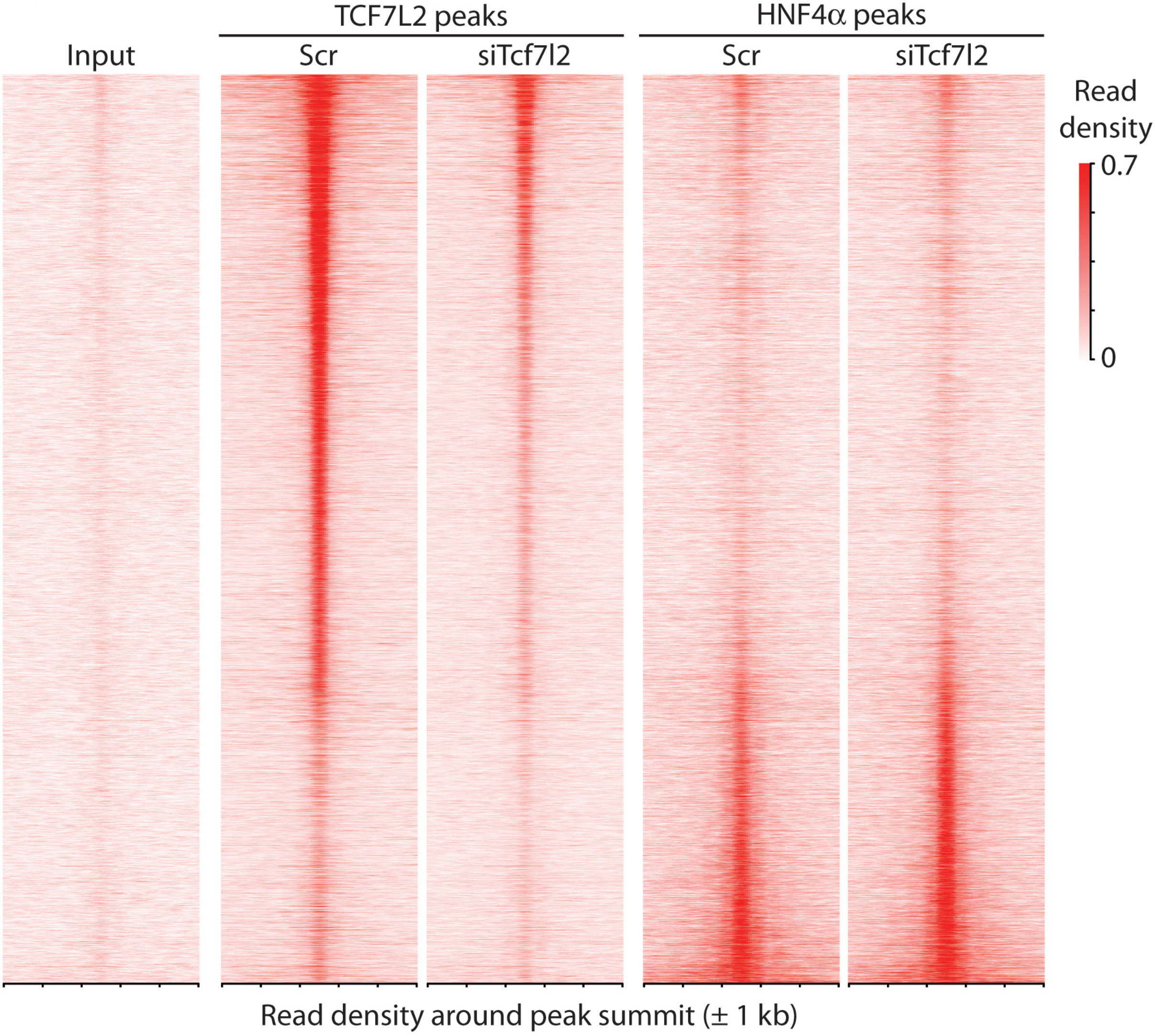

TCF7L2 peaks
HNF4α peaks
Input
Scr
siTcf7l2
Scr
siTcf7l2
Read density
0.7
0
Read density around peak summit (± 1 kb)

Figure S10

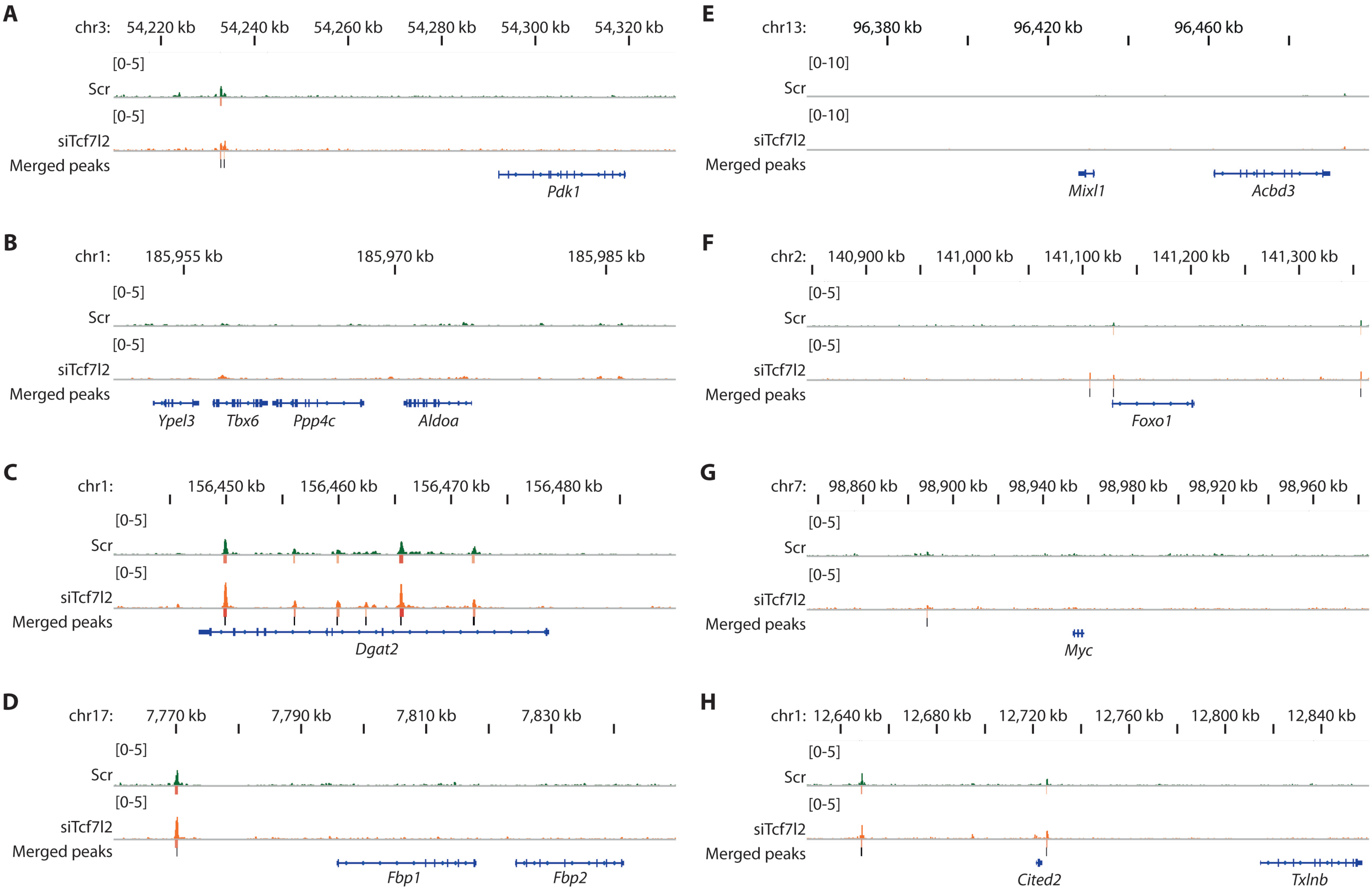
A
chr3: 54,220 kb 54,240 kb 54,260 kb 54,280 kb 54,300 kb 54,320 kb
[0-5]
Scr
[0-5]
siTcf7l2
Merged peaks
Pdk1
B
chr1: 185,955 kb 185,970 kb 185,985 kb
[0-5]
Scr
[0-5]
siTcf7l2
Merged peaks
Ypel3
Tbx6
Ppp4c
Aldoa
C
chr1: 156,450 kb 156,460 kb 156,470 kb 156,480 kb
[0-5]
Scr
[0-5]
siTcf7l2
Merged peaks
Dgat2
D
chr17: 7,770 kb 7,790 kb 7,810 kb 7,830 kb
[0-5]
Scr
[0-5]
siTcf7l2
Merged peaks
Fbp1
Fbp2
E
chr13: 96,380 kb 96,420 kb 96,460 kb
[0-10]
Scr
[0-10]
siTcf7l2
Merged peaks
Mixl1
Acbd3
F
chr2: 140,900 kb 141,000 kb 141,100 kb 141,200 kb 141,300 kb
[0-5]
Scr
[0-5]
siTcf7l2
Merged peaks
Foxo1
G
chr7: 98,860 kb 98,900 kb 98,940 kb 98,980 kb 98,920 kb 98,960 kb
[0-5]
Scr
[0-5]
siTcf7l2
Merged peaks
Myc
H
chr1: 12,640 kb 12,680 kb 12,720 kb 12,760 kb 12,800 kb 12,840 kb
[0-5]
Scr
[0-5]
siTcf7l2
Merged peaks
Cited2
Txlnb

Figure S11

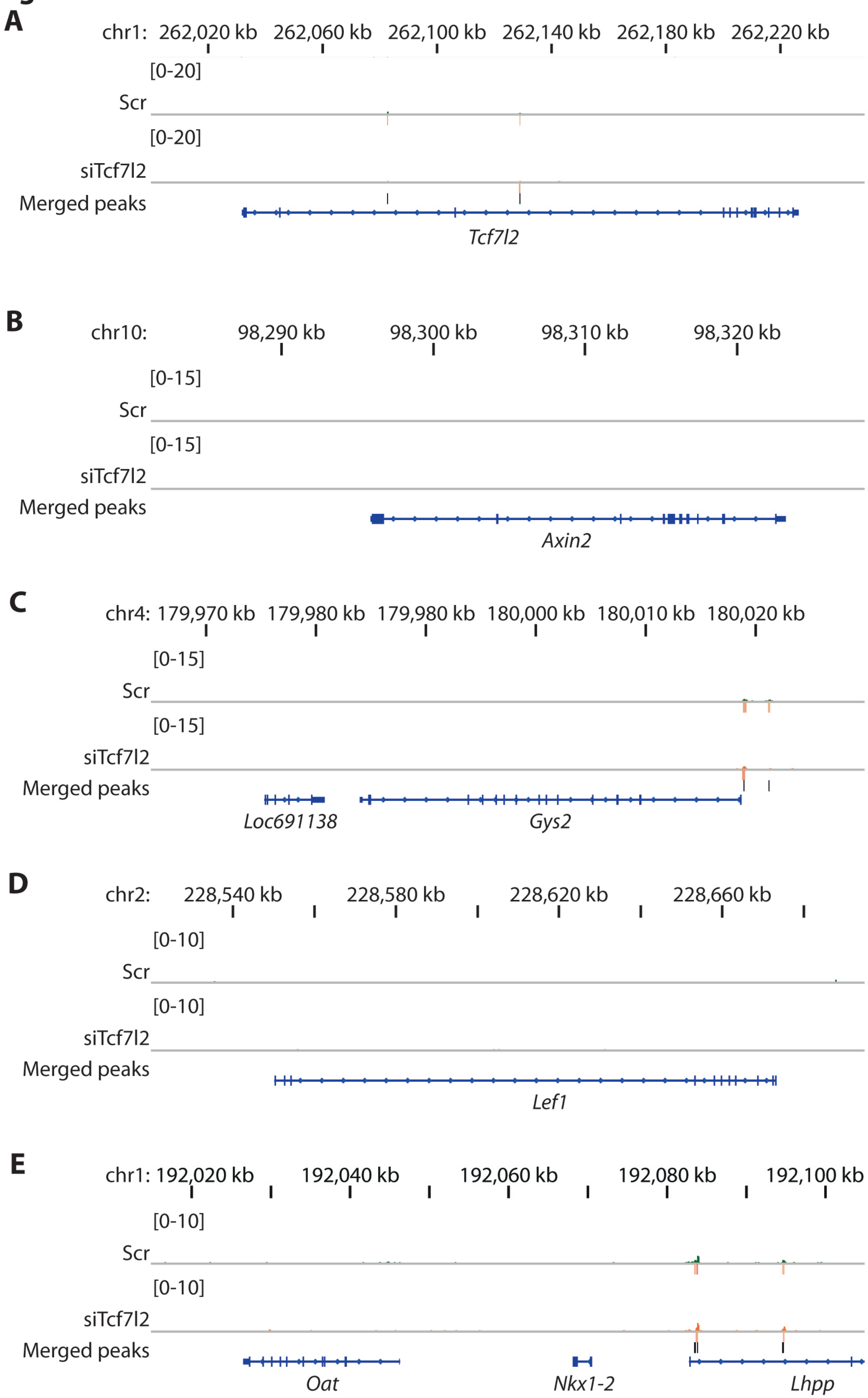
A
chr1: 262,020 kb 262,060 kb 262,100 kb 262,140 kb 262,180 kb 262,220 kb
[0-20]
Scr
[0-20]
siTcf7l2
Merged peaks
Tcf7l2
B
chr10: 98,290 kb 98,300 kb 98,310 kb 98,320 kb
[0-15]
Scr
[0-15]
siTcf7l2
Merged peaks
Axin2
C
chr4: 179,970 kb 179,980 kb 179,980 kb 180,000 kb 180,010 kb 180,020 kb
[0-15]
Scr
[0-15]
siTcf7l2
Merged peaks
Loc691138
Gys2
D
chr2: 228,540 kb 228,580 kb 228,620 kb 228,660 kb
[0-10]
Scr
[0-10]
siTcf7l2
Merged peaks
Lef1
E
chr1: 192,020 kb 192,040 kb 192,060 kb 192,080 kb 192,100 kb
[0-10]
Scr
[0-10]
siTcf7l2
Merged peaks
Oat
Nkx1-2
Lhpp

Figure S12

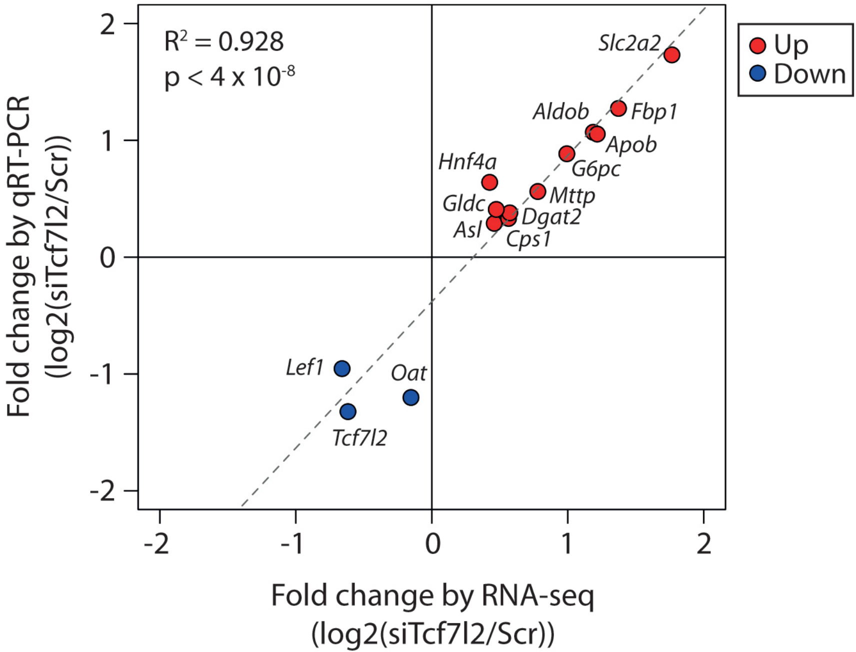

R² = 0.928
p < 4 x 10-8
Up
Down
Slc2a2
Aldob
Fbp1
Apob
Hnf4a
G6pc
Mttp
Gldc
Dgat2
Asl
Cps1
Lef1
Oat
Tcf7l2
2
1
0
-1
-2
-2
-1
0
1
2
Fold change by qRT-PCR
(log2(siTcf7l2/Scr))
Fold change by RNA-seq
(log2(siTcf7l2/Scr))
